# MicroRNA expression in multiple myeloma is associated with genetic subtype, isotype and survival

**DOI:** 10.1186/1745-6150-6-23

**Published:** 2011-05-18

**Authors:** Jianxiang Chi, Erica Ballabio, Xiao-He Chen, Rajko Kušec, Steve Taylor, Deborah Hay, Daniela Tramonti, Nigel J Saunders, Timothy Littlewood, Francesco Pezzella, Jacqueline Boultwood, James S Wainscoat, Christian SR Hatton, Charles H Lawrie

**Affiliations:** 1 Nuffield Department of Clinical Laboratory Sciences, University of Oxford, John Radcliffe Hospital, Oxford, UK; 2 The Center for the Study of Haematological Malignancies, Nicosia, Cyprus; 3 Dubrava University hospital and Zagreb School of Medicine, University of Zagreb, Croatia; 4 Computational Biology Research Group, Weatherall Institute of Molecular Medicine, University of Oxford, Oxford, UK; 5 Department of Haematology, John Radcliffe Hospital, Oxford, UK; 6 Sir William Dunn School of Pathology, University of Oxford, Oxford, UK

## Abstract

**Background:**

MicroRNAs are small RNA species that regulate gene expression post-transcriptionally and are aberrantly expressed in many cancers including hematological malignancies. However, the role of microRNAs in the pathogenesis of multiple myeloma (MM) is only poorly understood. We therefore used microarray analysis to elucidate the complete miRNome (miRBase version 13.0) of purified tumor (CD138^+^) cells from 33 patients with MM, 5 patients with monoclonal gammopathy of undetermined significance (MGUS) and 9 controls.

**Results:**

Unsupervised cluster analysis revealed that MM and MGUS samples have a distinct microRNA expression profile from control CD138^+ ^cells. The majority of microRNAs aberrantly expressed in MM (109/129) were up-regulated. A comparison of these microRNAs with those aberrantly expressed in other B-cell and T-cell malignancies revealed a surprising degree of similarity (~40%) suggesting the existence of a common lymphoma microRNA signature. We identified 39 microRNAs associated with the pre-malignant condition MGUS. Twenty-three (59%) of these were also aberrantly expressed in MM suggesting common microRNA expression events in MM progression. MM is characterized by multiple chromosomal abnormalities of varying prognostic significance. We identified specific microRNA signatures associated with the most common IgH translocations (t(4;14) and t(11;14)) and del(13q). Expression levels of these microRNAs were distinct between the genetic subtypes (by cluster analysis) and correctly predicted these abnormalities in > 85% of cases using the support vector machine algorithm. Additionally, we identified microRNAs associated with light chain only myeloma, as well as IgG and IgA-type MM. Finally, we identified 32 microRNAs associated with event-free survival (EFS) in MM, ten of which were significant by univariate (logrank) survival analysis.

**Conclusions:**

In summary, this work has identified aberrantly expressed microRNAs associated with the diagnosis, pathogenesis and prognosis of MM, data which will prove an invaluable resource for understanding the role of microRNAs in this devastating disease.

**Reviewers:**

This article was reviewed by Prof. Neil Smalheiser, Prof. Yuriy Gusev, and an unknown reviewer.

## Background

Multiple myeloma (MM) is a plasma cell (PC) malignancy with an annual incidence of over 14,000 cases in the US alone. MM is essentially an incurable disease with a median survival of ~3 years that accounts for nearly 2% of deaths from cancer and about 20% of deaths from hematological cancers [1]. Newer therapies, however, are resulting in improvements in the median survival [2]. Recent advances in molecular and genetic research have lead to the realization that MM, although defined histologically as a single entity, encompasses a wide range and frequently complex mixture of genomic abnormalities which differ in both their molecular pathogenesis and prognostic significance. The recent discovery of short non-coding RNA molecules that regulate gene expression post-transcriptionally, known as microRNAs, represent yet another level of complexity in our understanding of gene regulation and as such could further our understanding of the pathogenesis of MM.

MicroRNAs have been demonstrated to have diagnostic and prognostic potential in cancer [3-7] and it has been suggested that microRNA expression profiling can distinguish cancers according to both the cellular nature and the developmental stage of the tumor with a greater degree of accuracy than traditional gene expression analysis [8]. There is increasingly strong evidence that microRNAs are involved in the pathogenesis of many cancers including B and T cell lymphomas [9,10]. There is however little known about the role that microRNAs play in MM.

Therefore we undertook a comprehensive study using microarray technology to elucidate the complete miRNome (miRBase version 13.0) of purified tumor (CD138^+^) cells from the bone marrow of 33 MM and 5 MGUS patients (and 9 controls). In order to investigate microRNA expression in different genetic subtypes MM cases were classified cytogenetically by FISH. These data were then correlated with genetic subtype and clinical parameters.

## Results

### Most aberrantly expressed microRNAs associated with MM are up-regulated

**Figure 1:**
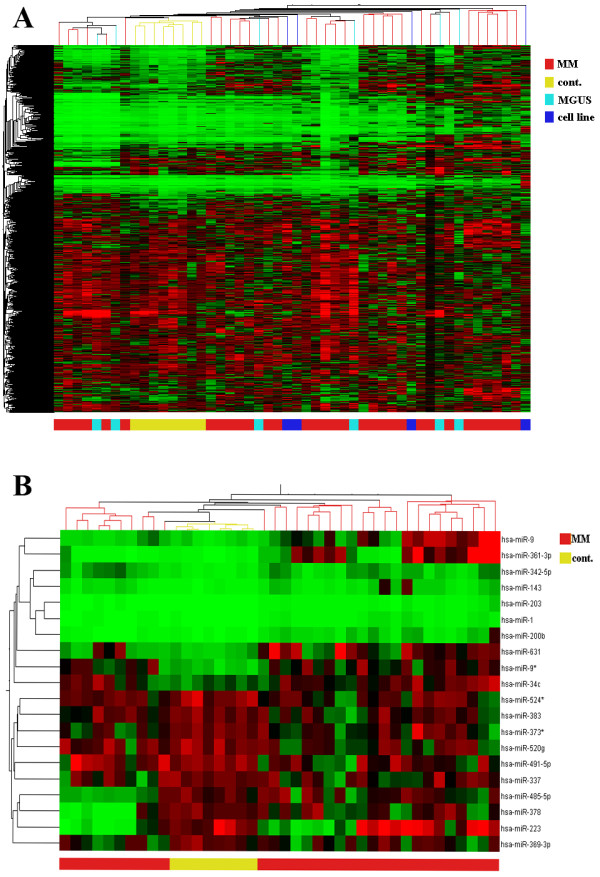
**The microRNA expression profile of MM is distinct from counterpart normal plasma cells**. (A) Unsupervised cluster analysis of microRNA expression data for MM (n = 33), control (n = 9) and MGUS (n = 5) samples. (B) Heat map depicting expression levels of 10 most discriminatory up- and down-regulated microRNAs (Table 1) between MM and control samples.

We elucidated the complete (miRBase v.10.1) microRNA profile of CD138^+ ^plasma B-cells from bone marrow of 33 MM patients, 5 MGUS, 9 controls, and 4 well established MM cell lines (NCI-H929, JJN3, Thiel and RPMI-8226). Unsupervised cluster analysis revealed that MM samples and MM cell lines have a distinct microRNA profile from counterpart controls (Figure 1A). Furthermore, although MGUS samples did not cluster together, they had a microRNA profile more similar to MM samples (and cell lines) than controls.

**Figure 2:**
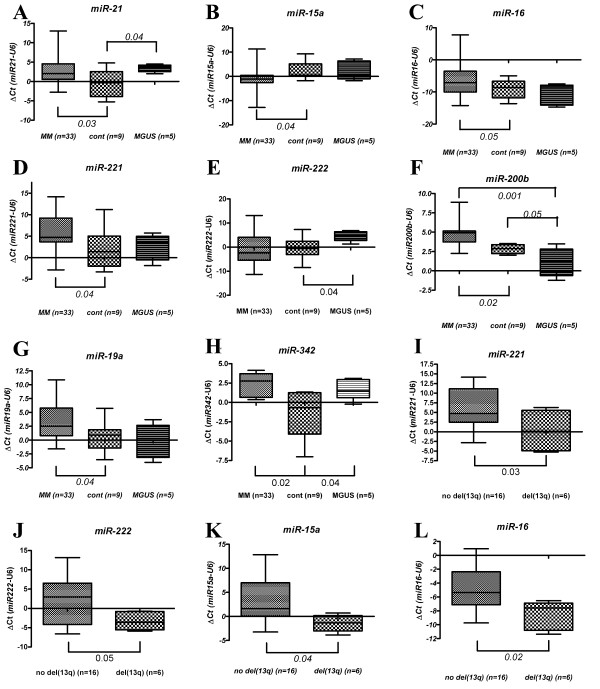
**Validation of microarray data by qRT-PCR**. Expression levels of (A) *miR-21*, (B) *miR-15a*, (C) *miR-16*, (D) *miR-221*, (E) *miR-222*, (F) *miR-200b*, (G) *miR-19a *and (H) *miR-342 *in MM (n = 33), control (n = 9) and MGUS (n = 5) samples measured by qRT-PCR. Expression levels of (I) *miR-221*, (J) *miR-222*, (K) *miR-15a *and (L) *miR-16 *in MM patients with and without del(13q). *P *values were calculated by Mann-Whitney independent *t*-test.

To identify microRNAs that are aberrantly expressed in MM patient samples we compared expression levels with controls by ANOVA. This resulted in the identification of 129 microRNAs (*P *< 0.05), only 20 (15%) of which were down-regulated in MM samples (Table 1). Of these 29 and 24 were previously identified as being aberrantly expressed in MM by the studies of Zhou *et al *[11] and Pichiorri *et al *[12] respectively. In order to validate the microarray data, eight microRNAs chosen for their previous association with MM or other hematological malignancies were measured by qRT-PCR. These data were consistent with the microarray results (Figure 2). Many microRNAs are encoded in clusters, and members of these clusters often exhibit the same pattern of expression [13]. A lower proportion of MM-associated microRNAs (42/109 (38%); Table 1) were encoded in clusters than generally observed (215/474 (45%)) (source- http://www.diana.pcbi.upenn.edu/cgi-bin/miRGen/v3/Cluster.cgi).

**Table 1:** MicroRNAs differentially expressed (*P *< 0.05) between MM (n = 33) and controls (n = 9) depicting chromosomal location and where relevant their expression within a microRNA cluster.

Order	microRNA	adj. *P *value	Fold change	Chromosome	Cluster
1	*miR-1*	5.41E-06	3.15	20q13.33/18q11.2	1-1, 133a
2	*miR-203*	2.09E-05	2.64	14q32.33	-
3	*miR-342*	2.25E-05	3.94	14q32.2	-
4	*miR-631*	2.25E-05	4.24	15q24.2	-
5	*miR-200a*	3.17E-05	5.50	12p13.31	141-200c
6	*miR-34c*	5.95E-05	3.31	11q33.1	34b-34c
7	*miR-361*	5.95E-05	12.66	Xq21.2	-
8	miR-9*	7.64E-05	4.48	1q22/5q14.3/15q26.1	-
9	*miR-200b*	8.52E-05	4.97	12p13.31	141-200c
10	*miR-9*	8.52E-05	5.07	1q22/5q14.3/15q26.1	-
11	*miR-151*	1.19E-04	2.67	8q24.3	-
12	***miR-218***	1.42E-04	3.96	4p15.31/5q34	-
13	*miR-28-3p*	1.90E-04	2.52	3q28	-
14	***miR-200c***	2.10E-04	5.26	12p13.31	141-200c
15	***miR-21***	2.72E-04	2.45	17q23.1	-
16	*miR-378*	2.72E-04	2.96	5q33.1	-
17	*miR-548d-5p*	2.72E-04	3.10	8q24.13/17q24.2	-
18	*miR-621*	2.72E-04	9.64	13q14.11	-
19	*miR-140-5p*	3.91E-04	2.78	16q22.1	-
20	*miR-634*	4.77E-04	2.25	17q24.2	-
21	*miR-616*	6.16E-04	2.69	12q13.3	-
22	***miR-130a***	7.81E-04	2.48	11q12.1	-
23	*miR-593*	7.81E-04	3.29	7q32.1	-
24	*miR-708*	7.81E-04	3.32	11q14.1	-
25	miR-200a*	7.81E-04	4.08	12p13.31	141-200c
26	*miR-340*	9.03E-04	2.41	5q35.3	-
27	*miR-760*	1.12E-03	2.44	1p22.1	-
28	*miR-885-3p*	1.39E-03	2.55	3p25.3	-
29	*miR-590-3p*	1.42E-03	3.50	7q11.23	-
30	*miR-885-5p*	1.61E-03	3.34	3p25.3	-
31	***miR-221***	2.36E-03	2.85	Xp11.3	221-222
32	*miR-7*	2.36E-03	3.66	9q21.32/15q26.1/19p13.3	-
33	***miR-188-5p***	2.94E-03	2.18	Xp11.23	532-660
34	*miR-338*	2.94E-03	2.35	17q25.3	338-657
35	*miR-222*	2.94E-03	2.47	Xp11.3	221-222
36	*miR-99a*	2.94E-03	3.47	19q13.33	99b-125a
37	*miR-891a*	3.06E-03	2.31	Xq27.3	890-891
38	*miR-452*	3.77E-03	2.57	Xq28	452-224
39	*miR-98*	4.09E-03	3.04	Xp11.22	98-let-7f
40	*miR-629*	4.09E-03	3.14	15q23	-
41	*miR-515-3p*	4.11E-03	5.90	Xq27.3	-
42	***miR-192***	4.20E-03	2.44	11q13.1	192-194
43	*miR-454*	4.36E-03	2.82	17q22	-
44	*miR-151-3p*	4.59E-03	2.22	8q24.3	-
45	*miR-141*	5.20E-03	3.37	12p13.31	141-200c
46	*miR-128b*	5.35E-03	2.25	2p21.3/3p22.3	-
47	*miR-1227*	5.64E-03	2.82	19p13.3	-
48	*miR-128a*	6.51E-03	2.82	2p21.3/3p22.3	-
49	*miR-205*	6.94E-03	3.15	1q32.2	-
50	*miR-27b*	7.30E-03	2.81	9q22.32	23b-24
51	*miR-608*	7.67E-03	2.38	10q24.31	-
52	*miR-432*	7.99E-03	2.41	14q32.2	337-136
53	*miR-220*	8.08E-03	2.86	Xq25	-
54	***miR-135a***	8.30E-03	2.80	3p21.1/12q23	-
55	*miR-34a*	8.42E-03	2.58	1p36.22	-
56	*miR-28*	8.72E-03	1.94	3q28	-
57	*miR-412*	9.41E-03	2.12	14q32.2	323-656
58	*miR-877*	9.84E-03	1.91	6p21.33	-
59	*miR-628-5p*	1.04E-02	2.09	15q21.3	-
60	*miR-532-3p*	1.06E-02	1.95	19q13.41	-
61	*miR-625*	1.15E-02	2.11	14q23.3	-
62	*miR-34b*	1.19E-02	2.07	11q33.1	34b-34c
63	*miR-31*	1.28E-02	2.62	9p21.3	-
64	***miR-181a***	1.46E-02	2.05	1q32.1/9q33.3	181a-181b
65	***miR-32***	1.46E-02	2.80	9q31.3	-
66	***miR-106b***	1.51E-02	2.48	7q22.1	106b-25
67	***miR-146a***	1.52E-02	2.14	5q33.3	-
68	*miR-210*	1.52E-02	2.87	11p15.5	-
69	*miR-499-5p*	1.54E-02	6.60	20q11.2	-
70	*miR-140*	1.58E-02	2.35	16q22.1	-
71	*miR-188*	1.68E-02	1.99	Xp11.23	532-660
72	*miR-610*	1.68E-02	2.54	11p14.1	-
73	***miR-27a***	1.70E-02	2.45	19p13.12	24-23a
74	*miR-142-5p*	1.70E-02	2.47	17q22	-
75	*miR-603*	1.75E-02	1.80	10p12.2	-
76	*miR-660*	1.75E-02	2.13	Xp11.23	532-502
77	*miR-19a*	1.75E-02	2.79	13q31.3/Xq26.2	17-92/106a-363
78	*miR-649*	1.78E-02	1.91	22q11.21	-
79	*miR-140-3p*	1.98E-02	2.12	16q22.1	-
80	*miR-300*	2.00E-02	2.30	14q32.31	543-655
81	***miR-335***	2.11E-02	2.11	7q32.2	-
82	*miR-206*	2.20E-02	1.84	6p12.2	206-133b
83	*miR-20b*	2.20E-02	2.18	13q31.3/Xq26.2	17-92/106a-363
84	*miR-130b*	2.25E-02	2.19	22q11.21	301b-130b
85	*miR-183*	2.40E-02	2.50	7q32.2	183-182
86	*miR-652*	2.44E-02	2.36	Xq22.3	-
87	*miR-133b*	2.44E-02	2.41	6p12.2	133b-206
88	miR-191*	2.56E-02	1.97	3p21.31	425-191
89	***miR-19b***	2.58E-02	2.62	13q31.3/Xq26.2	17-92/106a-363
90	*miR-212*	2.71E-02	1.97	17p13.3	212-132
91	*miR-194*	2.73E-02	2.09	1q41/11q13.1	215-194/192-194
92	***miR-100***	2.82E-02	2.82	11q24.1	100-let7a
93	*miR-1234*	2.88E-02	1.91	8q24.3	-
94	*miR-182*	2.96E-02	2.29	7q32.2	183-182
95	*miR-888*	3.28E-02	2.15	5q33.1	-
96	***miR-30e-5p***	3.55E-02	2.27	1p34.2	30e-30c
97	*miR-574*	3.60E-02	2.16	4p14	-
98	*miR-135b*	3.60E-02	2.31	1q32.1	-
99	***miR-125b***	3.60E-02	2.43	11q24.1/21q21	-
100	*miR-502*	3.77E-02	1.79	Xp11.23	500-502
101	*miR-320*	3.77E-02	1.74	8p21.3	-
102	*miR-421*	3.77E-02	1.76	Xq13.2	421-374b
103	*miR-129-3p*	3.77E-02	1.80	7q32.1/11p11.2	-
104	***miR-190b***	4.03E-02	2.02	1q21.3	-
105	*miR-18a*	4.44E-02	2.21	13q31.3/Xq26.2	17-92/106a-363
106	*miR-549*	4.44E-02	2.25	15q25.1	-
107	***miR-338-5p***	4.45E-02	1.97	17q25.3	338-657
108	*miR-576-3p*	4.60E-02	4.80	4q25	-
109	*miR-133a*	4.62E-02	2.32	6p12.2	133b-206
-1	miR-373*	1.39E-03	-1.82	19q13.42	371-373
-2	miR-378*	2.43E-03	-3.34	5q33.1	-
-3	*miR-143*	8.83E-03	-1.80	5q33.1	-
-4	***miR-15a***	1.51E-02	-1.67	13q14	15a-16
-5	*miR-337*	1.52E-02	-1.65	14q32.2	493-432
-6	***miR-223***	1.54E-02	-3.82	Xq12	-
-7	*miR-369-3p*	1.55E-02	-1.83	14q32.31	323-656
-8	*miR-520g*	1.58E-02	-1.90	19q13.42	517-518
-9	*miR-485-5p*	1.75E-02	-3.18	19q13.42	517-518
-10	miR-524*	1.86E-02	-1.83	19q13.42	517-518
-11	*miR-520h*	2.20E-02	-1.98	19q13.42	517-518
-12	*miR-516-3p*	2.20E-02	-1.81	19q13.42	517-518
-13	*miR-519d*	2.20E-02	-1.78	19q13.42	517-518
-14	*miR-371-3p*	2.34E-02	-3.58	19q13.42	517-518
-15	*miR-455*	2.88E-02	-1.88	9q32	-
-16	*miR-520b*	2.96E-02	-1.85	19q13.42	517-518
-17	*miR-518d*	3.28E-02	-2.09	19q13.42	517-518
-18	*miR-624*	3.28E-02	-1.56	14q12	-
-19	*miR-296*	3.59E-02	-1.85	21q22.12	-
-20	***miR-16***	4.69E-02	-1.83	13q14	15a-16

Positive values are up-regulated in MM and negative values down-regulated. MicroRNAs in common with Zhou *et al *[11] are denoted in red font and Pichiorri *et al *[12] in bold font.

Expression levels of the 129 MM-associated microRNAs were distinct between MM and control samples (Additional file 1 Figure S1). Indeed using only expression levels of the ten most discriminatory (by *P*-value) up- and down-regulated microRNAs distinguished samples by cluster analysis (Figure 1B) and correctly predicted samples as either MM or control for 36/41 (88%) samples using the leave-one-out cross-validation support vector machine (SVM) algorithm.

### The microRNA profile of MGUS patient samples is distinct from MM and controls

MGUS is generally believed to represent a pre-malignant form of MM [14]. To examine microRNA expression in this condition we initially compared the profile of PC cells from MGUS patients with that of controls. Thirty-nine microRNAs were identified as being aberrantly expressed in MGUS samples (*P *< 0.05; Additional file 1 Table S3); 28 microRNAs were up-regulated and 11 down-regulated. Nine of these were consistent with a previous study [12], including up-regulation of *miR-21*, the most up-regulated microRNA identified (10.3 fold change; Figure 2A), *miR-222 *and *miR-342 *(Figure 2E, H respectively), and down-regulation of *miR-200b *(Figure 2F). Expression levels of MGUS-associated microRNAs distinguished between MGUS and controls by cluster analysis (Additional file 1 Figure S2), and correctly predicted 12/13 (92%) the presence of this disorder by SVM analysis.

### Different genetic subtypes of MM have distinct microRNA expression profiles

Recently it has become increasingly clear that MM displays an enormous genomic complexity that underlies the clinical heterogeneity observed with this disease. The presence of recurrent chromosomal abnormalities such as IgH translocations and del(13q) have been shown to have prognostic significance in multiple studies and as a consequence cytogenetic classification is increasingly being used to stratify MM patients in clinical practice although not yet routinely [15,16]. In order to investigate microRNA expression in the different genetic subtypes of MM, twenty-six cases (for which there was sufficient material) were cytogenetically classified using the micro-FISH technique [17]. Individual patient karyotype data are summarized in Additional file 1 Table S1. Fourteen of 26 (54%) of MM cases harbored an IgH translocation and 7/26 (27%) exhibited the 13q (*RB*) deletion. Of the translocation group eight (57%) displayed the t(11;14) translocation, three (21%) cases the t(4;14) translocation, and three cases had an IgH translocation with an un-known (tested) fusion partner. Ten (38%) MM patients had no detectable chromosomal abnormality using our FISH probe sets, although it is probable that these cases had undetected abnormalities.

**Figure 3:**
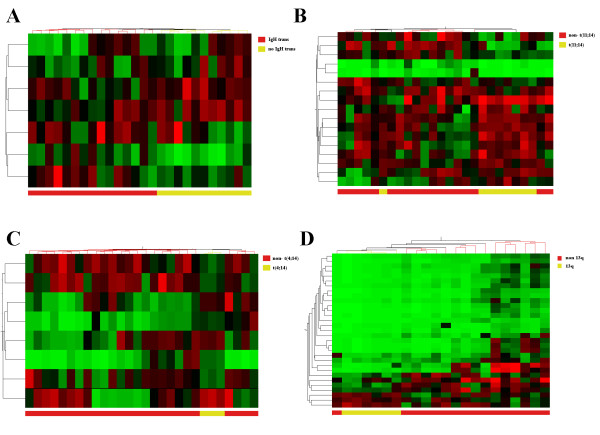
**MicroRNA expression can distinguish between different genetic subtypes of MM**. Cluster analysis heat maps depicting expression levels of microRNAs associated with (A) IgH translocation, (B) t(11;14), (C) t(4;14) and (D) del(13q).

The majority of MM tumors harbor IgH translocations, the most common fusion partners being 11q13 (*CCND1*) and 4p16 (*FGFR3*) [18]. A comparison between cases containing an IgH translocation with those that did not identified seven differentially expressed (P < 0.05) microRNAs (Table 2). Expression levels were distinct in MM cases containing the IgH translocation (Figure 3A), and predicted 24/26 (92%) of cases by SVM analysis. Seventeen microRNAs were identified as being associated with the most common IgH translocation event, t(11;14) (*IgH*:*CCND1*) (P < 0.05; Table 2), four of which (*miR-375, miR-650*, *miR-193a *and *miR-582*) were consistent with other studies [19,20]. Expression levels of these microRNAs distinguished cases with t(11;14) (Figure 3B), and correctly predicted the presence of this translocation in 24/26 (92%) of cases by SVM analysis. Three cases in our cohort had the t(4;14) (*IgH:FGFR3*) translocation. Eight microRNAs were associated with this translocation (P < 0.05; Table 2), three of which (*miR-99b, miR-342 *and *miR-214*) were previously identified [19,20]. Expression levels of these microRNAs distinguished cases with the t(4;14) translocation (Figure 3C) and correctly predicted all cases containing this translocation. Interestingly, four of the six up-regulated microRNAs are encoded on either chromosome 4 or 14 (*miR-376a *(14q33.21), *miR-342 *(14q32.2), *miR-574 *(4p14) and *miR-577 *(4q26)), and of the two microRNAs (*miR-520c *and *miR-376a*) aberrantly expressed in both t(4;14) and t(11;14) cases, the latter is encoded proximal to the IgH breakpoint region at 14q32.

**Table 2:** MicroRNAs associated (*P *< 0.05) with different genetic subtypes depicting chromosomal location.

Cytogenetic group	microRNA	Fold change	*P*-value	Chromosome
**IgH trans (n = 14)**	*miR-590*	3.28	1.62E-02	7q11.23
	*miR-886*	1.63	1.46E-02	5q31.1
	*miR-33b*	1.48	2.46E-02	17p11.2
	*miR-184*	-1.43	3.87E-02	15q25.1
	*miR-139*	-1.44	1.47E-02	11q13.4
	*miR-508*	-1.63	4.55E-03	Xq27.3
	*miR-579*	-1.66	2.43E-02	5p13.3
**t(11;14) (n = 8)**	miR-202*	2.16	3.10E-03	10q26.3
	*miR-520c*	2.05	1.26E-03	19q13.42
	*miR-890*	2.04	4.39E-02	Xq27.3
	***miR-582***	1.91	4.06E-02	5q12.1
	*miR-122a*	1.88	1.40E-02	3p21.31
	miR-526b*	1.86	1.09E-04	19q13.42
	***miR-375***	-1.74	2.04E-02	2q35
	*miR-543*	-1.59	3.46E-02	14q33.21
	***miR-650***	-1.58	4.00E-02	22q11.22
	***miR-193a***	-1.51	1.35E-02	17q11.2
	*miR-147b*	-1.46	2.76E-02	15q21.1
	*miR-526a*	-1.51	4.68E-02	19q13.42
	*miR-542*	-1.56	1.81E-02	Xq26.3
	*miR-301*	-2.22	4.80E-02	17q22
	*miR-26b*	-2.62	3.81E-02	2q35
	*miR-376a*	-2.63	2.68E-02	14q33.21
	*miR-21*	-2.82	5.19E-03	17q23.1
**t(4;14) (n = 3)**	*miR-376a*	3.48	1.23E-02	14q33.21
	*miR-574*	3.11	1.23E-02	4p14
	***miR-99a***	2.53	1.23E-02	21q21.1
	***miR-214***	2.12	2.77E-02	1q24.3
	*miR-577*	1.97	3.85E-02	4q26
	***miR-342***	1.79	3.50E-02	14q32.2
	*miR-935*	-2.07	3.55E-02	19q13.42
	*miR-520c*	-2.23	3.85E-02	19q13.42
**13q(del) (n = 6)**	***miR-221***	-9.71	1.18E-04	Xp11.3
	*miR-222*	-9.50	1.78E-03	Xp11.3
	***let-7b***	-5.15	3.38E-03	22q13.31
	*let-7a*	-4.92	3.72E-03	9q22.32/11q24.1
	***let-7c***	-4.86	6.70E-03	21q21.1
	***miR-15a***	-4.42	6.39E-03	13q14
	***miR-20a***	-4.41	1.47E-03	13q31
	*miR-107*	-4.40	3.23E-03	10q23.31
	*miR-26a*	-4.37	3.26E-03	3p22.2/12q14.1
	*miR-103*	-4.30	8.98E-03	5q34/20p13
	*miR-142*	-4.13	1.15E-03	17q22
	*miR-195*	-3.97	6.98E-03	17p13.1
	*miR-146a*	-3.92	1.46E-03	5q33.3
	*miR-23a*	-3.80	6.44E-03	19p13.12
	*miR-92a*	-3.77	3.57E-03	13q31
	*miR-27a*	-3.58	2.25E-03	19p13.12
	*miR-361*	-3.46	4.19E-03	Xq21.2
	*miR-145*	-3.43	7.10E-03	5q32
	*miR-92b*	-3.37	9.82E-03	13q31
	***miR-19a***	-3.36	5.57E-03	13q31
	miR-199a*	-2.93	5.93E-03	19p13.2/1q24.3
	*miR-34a*	-2.89	2.56E-03	1p36.22
	*miR-188*	-2.79	8.43E-03	Xp11.23
	***miR-196a***	-2.40	5.60E-03	17q21.32
	*miR-485*	-2.33	4.18E-03	19q13.42
	***miR-16***	-2.23	7.79E-03	13q14
	*miR-493*	-1.89	2.46E-03	14q32.2

Comparison between MM cases with (positive fold change) and without (negative fold change) chromosomal abnormality as indicated in text. MicroRNAs also identified by Gutiérrez *et al *[19] or Lionetti *et al *[20] are depicted in bold type.

A comparison between microRNA expression in del(13q) MM patients with patients not harboring this deletion identified 28 microRNAs by ANOVA (*P *< 0.05; Table 2). Expression levels of these microRNAs distinguished cases that were del(13q) by cluster analysis (Figure 3D), and correctly predicted its presence in 22/26 (85%) of cases by SVM analysis. Eight of these microRNAs were consistent with research by Gutiérrez and colleagues [19], including down-regulation of *miR-15a *and *miR-16*, encoded within the 13q14 locus (Figure 2K and 2L respectively).

### MicroRNA expression is associated with clinical parameters and survival

The most common paraprotein isotypes associated with MM are IgG (60%) and IgA (24%) [21]. It has been reported that IgA myeloma has a poorer prognostic outcome than patients with IgG myeloma [22], although it should be noted that we (data not shown) and others [23] did not find any survival differences between isotypes. Consequently we compared expression levels between thirteen cases of IgG-type MM with eight cases of IgA-type. This resulted in the identification of twenty-one differentially expressed microRNAs (*P *< 0.05; Additional file 1 Table S5). Expression levels of these microRNAs were distinct between IgA and IgG MM cases (Additional file 1 Figure S4), and correctly predicted isotype for 20/21 (95%) of cases by SVM analysis.

Approximately 10% of MM patients secrete only light chains instead of the complete immunoglobulin molecule [21]. So called "light chain only myeloma" or "Bence Jones myeloma" had been reported to have a poorer prognostic outcome [22,24] although a more recent (and comprehensive) study found no such difference [23]. In our cohort eight MM cases had light chain only myeloma. Twenty-seven microRNAs were identified as being differentially expressed compared to non-light chain only MM cases (Additional file 1 Table S6). Expression levels of these microRNAs correctly predicted light-chain only myeloma in all cases.

**Figure 4:**
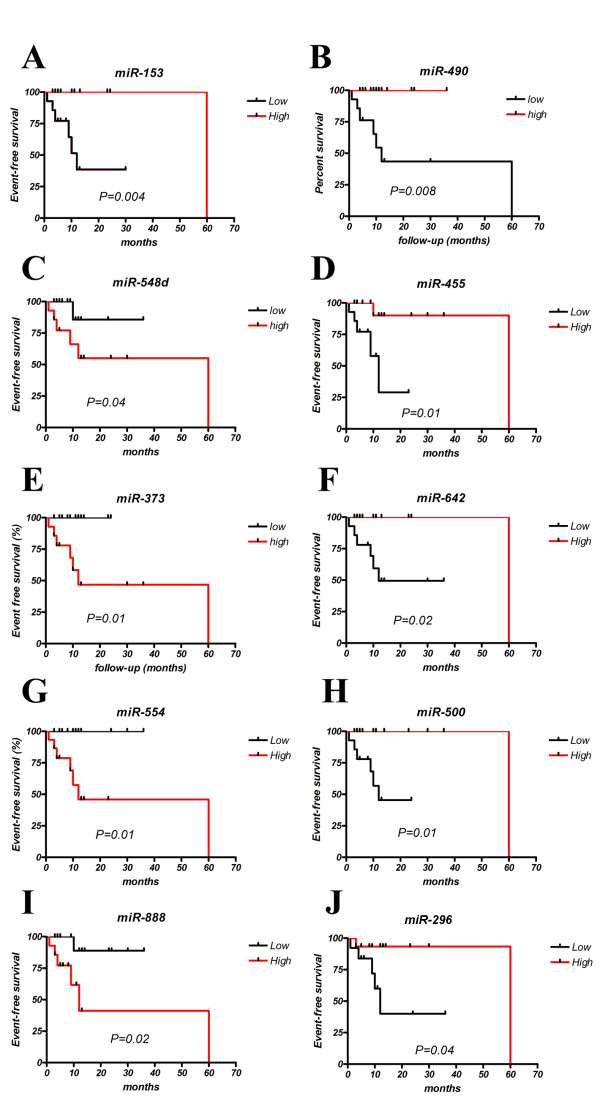
**Kaplan-Meier survival curves of EFS in MM patients based on high or low (median) expression levels of microRNAs (*P *< 0.05)**. Curves were compared by univariate (logrank) analysis. (A) *miR-153*, (B) *miR-490*, (C) *miR-548d*, (D) *miR-455*, (E) *miR-373*, (F) *miR-642*, (G) *miR-554*, (H) *miR-500*, (I) *miR-888 *and (J) *miR-575*.

As we had successfully identified microRNAs associated with clinical outcome in diffuse large B-cell lymphoma [7], we assessed differences in microRNA expression between poor outcome and good outcome in MM cases, using event-free survival (EFS) as our clinical criterion (median follow-up = 20 months). Samples from patients with event-free survival (n = 21) were contrasted with those from patients who had relapsed or died in this interval (n = 7). Thirty-two microRNAs were identified as being differentially expressed (P < 0.05) (Additional file 1 Table S7). These microRNAs correctly predicted relapse in 24/28 (86%) of cases by SVM analysis. To investigate the effect of individual microRNA expression levels on clinical outcome we carried out log-rank (univariate) survival analysis and found that low expression of *miR-153, miR-490*, *miR-455*, *miR-642*, *miR-500*, and *miR-296*, and high expression of *miR-548d, miR-373, miR-*554 and *miR-888 *were associated (*P *< 0.05) with EFS (Figure 4).

## Discussion

In this study we used microarray technology to elucidate the complete miRNome (miRBase version 13.0) of purified PCs from 33 MM and 5 MGUS patients and compared their expression with counterpart normal PCs. One hundred and twenty-nine microRNAs were identified as being aberrantly expressed in MM. Thirty-nine (30%) of these were previously reported to be aberrantly expressed in MM by Pichiorri *et al *[12] and/or Zhou *et al *[11]. The reason for these discrepancies probably reflects the use of differing statistical, microarray and sampling methods. Of note the former study only examined 10 MM and 4 controls, whilst the latter study included only 2 controls for analysis. Consistent with the findings of Pichiorri and colleagues we found that *miR-21 *was up-regulated in both MM and MGUS as were all seven microRNAs encoded by *miR-17-92 *cluster (average fold-change = 2.1; range 1.75-2.78). However unlike that study, although *miR-32 *and *miR-181a *were identified as being up-regulated by microarray analysis, we failed to find any significant difference in expression levels by qRT-PCR (data not shown). Surprisingly 10 of the 20 down-regulated microRNAs identified in the current study are encoded by the *miR-371-1323 *cluster located at 19q13.42. This cluster consists of 49 microRNAs, 39 of which were present on our microarray. All members of this cluster were down-regulated (average 1.6-fold; range 1.1-2.5). Interestingly, members of this cluster (*miR-373 *and *miR-520c*) have been demonstrated to be associated with tumor invasion and metastasis [25].

The vast majority (109/129 (85%)) of microRNA aberrantly expressed in MM were up-regulated, consistent with the findings of Zhou *et al *[11]. We [7,26] (and others[27]) found the same pattern in other B-cell malignancies. In contrast, we [10] (and others [28]) recently demonstrated that T-cell malignancies are associated with a global decrease in microRNA expression. This suggests a fundamental difference in microRNA biogenesis between B and T-cell malignancies. A possible explanation for this distinction comes from our previous observation that components of the microRNA biosynthetic pathway (i.e. the microprocessor complex (*DGCR8 *and *Drosha*)) are up-regulated in B-cell malignancies (including MM) but down-regulated in T-cell malignancies [29].

**Figure 5:**
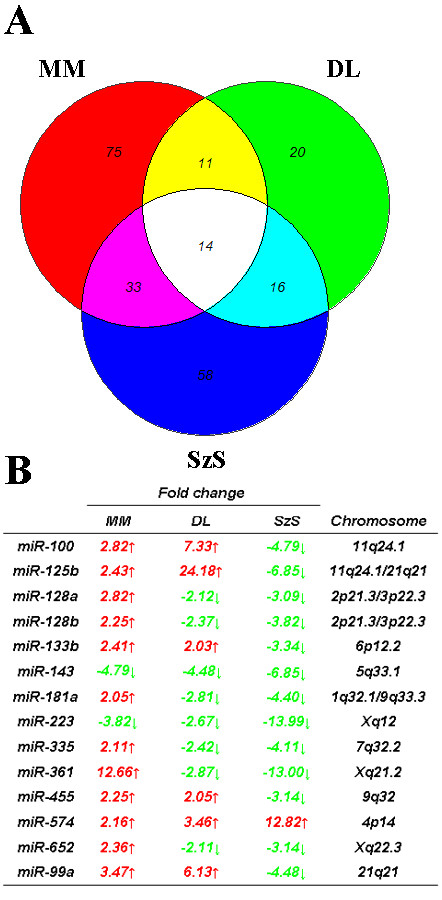
**MicroRNAs commonly dysfunctionally expressed in multiple lymphoma types**. (A) Venn diagram showing relationship between microRNAs aberrantly expressed in diffuse large B-cell lymphoma (DLBCL) [7], Sezary syndrome (SzS) [10] and multiple myeloma (MM) ((Table 1) (Additional file 1, Table S2)). (B) Tables of microRNAs aberrantly expressed in all lymphoma types depicting fold change relative to normal counterpart controls.

Using the same array as in this study, we previously identified 60 and 119 microRNAs aberrantly expressed in diffuse large B-cell lymphoma (DLBCL) [7] and Sézary syndrome (SzS) (T-cell lymphoma) respectively [10]. A comparison with MM-associated microRNAs revealed that 25/60 (42%) and 47/119 (40%) of microRNAs aberrantly expressed in DLBCL and SzS respectively were also aberrantly expressed in MM (Figure 5 andAdditional file 1 Table S2). Fourteen microRNAs were dysfunctionally expressed in all three lymphoma types with *miR-223 *and *miR-143 *commonly down-regulated and *miR-574 *commonly up-regulated. Although this analysis is by no means exhaustive this suggests the presence of a common lymphoma microRNA signature. Down-regulation of *miR-223 *also has been demonstrated in hepatocellular carcinoma [30], acute myeloid leukemia [31] and chronic lymphocytic leukemia [32,33], and shown to regulate the important hematopoietic regulator *LMO2 *[34] that is commonly expressed in lymphoma [35]. Similarly, *miR-143 *has been reported to be commonly down-regulated in hematological malignancy [36]. We are currently investigating expression of these microRNAs in a range of lymphoma types to see if their dysregulation is truly a common feature of lymphoma.

MGUS has been shown to consistently proceed the development of MM [37] and as such is considered to represent a pre-malignant model for MM pathogenesis. Twenty-three of the 39 (59%) microRNAs aberrantly expressed in MGUS were also aberrantly expressed in MM (Additional file 1 Table S3) suggesting common microRNA expression events in MM progression. Thirty-seven microRNAs were identified as being differentially expressed between MGUS and MM (Additional file 1 Table S4), only 9 of these were also differentially expressed in MGUS compared to controls (i.e. specific for MGUS) including *miR-21 *as previously identified [12]. The relationship between microRNAs dysfunctionally expressed in MGUS and MM are shown in Additional file 1 Figure S3. *miR-21 *over-expression has been linked to an anti-apoptotic phenotype and enhanced tumor growth [38,39] and as such may represent an important aberration in the early pathogenesis of MM.

Next we investigated the identity of microRNAs associated with the major genetic subtypes of MM. Two previous studies by Lionetti *et al *[20] and Gutiérrez *et al *[19] have also examined microRNA expression associated with recurrent chromosomal abnormalities in MM. Generally our findings were consistent with these studies although microRNAs associated with specific abnormalities did not always completely overlap. The cause for discrepancies between studies, including lack of concordance between the two previously published studies, again probably represents differences between microarray platforms, statistical techniques and samples used for analysis. For example the study of Gutiérrez *et al *considered cases in which del(13q) was the sole chromosomal abnormality and deregulated microRNAs were identified on the basis of their expression relative to control PCs, whereas our study and that of Lionetti *et al *considered the difference between MM cases with and without specific chromosomal abnormalities [19,20]. The latter study considered genetic abnormalities in the context of a gene-expression defined molecular translocation/cyclin (TC) classification system [20]. Such differences between studies are a reflection of the relative immaturity of the microRNA field that has only formally existed for the last 9 years, and is something that will surely be resolved in future years.

Interestingly, from the 28 microRNAs associated with the presence of del(13q), all seven microRNAs encoded by the *miR-17-92 *cluster (encoded at 13q31) were down-regulated (average 3.1 fold; range 1.2-4.1). Recent data suggests that unlike the characteristic del(13q) karyotype observed in chronic lymphocytic leukemia (CLL) defined by loss of the 13q14 locus, the minimal deleted region of 13q loss in MM extends from 13q14 to the 13q34 locus [40], consistent with down-regulation of the *miR-17-92 *cluster observed in these cases. Although down-regulation of the *miR-17-92 *cluster was also reported by Gutiérrez *et al *[19], this finding is counter-intuitive as members of this cluster are widely believed to function as oncogenes [41] whilst the presence of del(13q) is associated with an adverse prognostic outcome in MM [42]. A possible explanation for this apparent paradox is that although widely up-regulated in a wide range of cancers [9,26,43], the *miR-17-92 *cluster has been proposed to act as both tumor-suppressor or oncogene depending upon the cellular context [44-46]. Recently we reported that this cluster is down-regulated in T-cell lymphoma where it was shown to have tumor-suppressor properties [10]. Consequently, it is possible that the cluster is similarly acting as a tumor-suppressor in del(13q) MM cases although it should be noted that up-regulation of this cluster is associated with MM diagnosis (Table 1) and at least one member of this cluster (*miR-19*) was found to antagonize MM tumor development in a murine model [12].

We also report for the first time a correlation between microRNA expression and isotype in MM including the presence of light-chain only myeloma. Such information might be useful in providing an alternative clinical classification of new MM patients. Finally, we examined how the expression levels of individual microRNAs might influence clinical outcome of MM patients by correlating expression values with adverse effects using Kaplan-Meier survival analysis. We found that high expression of *miR-153*, *miR-490*, *miR-455*, *miR-642*, *miR-500*, and *miR-296 *were associated with better EFS in our cohort of patients whilst the opposite was true for *miR-548d, miR-373, miR-*554 and *miR-888*. Interestingly, the most discriminatory of these microRNAs (i.e. *miR-153*) has been shown to target *BCL2 *and *MCL1 *[47] and that over-expression of both of these are associated with poor prognostic outcome in MM [48]. Low expression of *miR-455 *has also been associated poor overall survival in endometrial serous adenocarcinoma [49], and low levels of *miR-296 *shown to be associated with increased prostate tumor growth and invasion properties via targeting of *HMGA *[50]. It should be noted however that these data need to be tested in an independent test cohort before any firm conclusions can be drawn about the prognostic ability of these microRNAs in MM patient survival, whilst the current results provide the experimental basis for future validation experiments.

## Conclusions

In summary, these results indicate that aberrant expression of microRNAs is a common feature of MM as well as pre-malignant MGUS, and that this phenomenon is associated with genetic and clinical subtype as well as clinical outcome. This data reinforces the complex nature of MM and hopefully will provide useful information in clarifying the molecular basis of this disease in future studies.

## Methods

### Patient samples and cell lines

Bone marrow samples were obtained from patients attending the Department of Haematology, John Radcliffe Hospital, Oxford, UK or Dubrava University hospital Zagreb, Croatia. Patients were diagnosed as MM (n = 33) or MGUS (n = 5) according to criteria described in the myeloma management guidelines [51]. The median age of patients was 67 yrs (range 43-89 yrs). Individual patient details are shown in Additional file 1 Table S1. Plasma cells (PC) were purified from bone marrow aspirates by positive immunomagnetic selection (CD138^+^) according to manufacturer's instructions (Miltenyi Biotec, Bisley, UK). PCs obtained from the bone marrow of nine healthy individuals were used as controls. The purity of PC samples was > 90% CD138^+ ^as measured by immunohistochemical staining.

Patient samples were collected in accordance with the Declaration of Helsinki and approved by the local ethics committee (Oxforshire Regional Ethics Committee; Ref no. 07/Q1606/25). Patients gave written informed consent for the sample collection.

MM cell lines, NCI-H929, JJN3, RPMI8226 and Thiel were routinely cultured in RPMI 1640 containing 10% fetal calf serum (Invitrogen, Paisley, UK). All cell lines were obtained from the DSMZ cell collection (Braunschweig, Germany) except Thiel which was a kind gift from Prof. Diehl (University of Cologne, Germany).

### Cytogenetic classification of myeloma samples

Purified MM patient PCs (~10^5 ^cells) were classified according to cytogenetic criteria using the micro-FISH technique as previously described [17]. The following probe sets obtained from Vysis (Abbott Diagnostics, Maidenhead, UK) were used for classification; D13S319 Spectrum-Orange/LSI-13q34-Spectrum-Green (del(13q) probe), IgH Spectrum-Green/FGFR3 Spectrum-Orange (t(4;14) fusion probe), IgH Spectrum-Green/CCND1 Spectrum-Orange (t(11;14) fusion probe), and IgH dual-color (Spectrum-Green/Spectrum-Orange) break-apart probe [52]. At least 100 nuclei were scored for each probe. A 20% cut-off was used for numerical abnormalities and 10% for fusions and break-apart probes as recommended by the European Myeloma Network FISH workshop guidelines http://www.myeloma-europe.org.

### RNA purification and microarray analysis

MicroRNA was isolated from samples (~10^6 ^cells) using Trizol (Invitrogen, Paisley, UK) and RNeasy columns as described by the manufacturer (Qiagen, Crawley, UK). MicroRNA (~500 ng) were labeled and hybridized to μRNA microarrays as previously described [26] using tonsillar material (pooled from twelve healthy individuals) as a common reference in a dye-balanced design. The arrays contained 655 human probes (miRBase v.10.1). Probe details can be found at http://www.microRNAworld.com.

Image analysis was carried out with BlueFuse software (BlueGnome, Cambridge, UK). Raw image data were global loess-normalized within arrays and normalized between arrays using the LIMMA package [53]. The normalized log ratios (average of four replicates per probe) were used for subsequent analysis in Genespring 7.2 (Agilent Technologies, CA, US). ANOVA analysis was used to identify microRNAs differentially expressed between sample types and *P *values were adjusted using the Benjamini-Hogberg correction method. Differentially expressed genes were tested for their ability to predict sample class using the leave-one-out cross-validation support vector machine (SVM) function in Genespring (Polynomial Dot Product (Order 1) Kernel Function. Diagonal Scaling Factor: 0). All microarray data was MIAME compliant and raw data has been deposited in the GEO database (Accession series # GSE243371).

### Quantitative RT-PCR (qRT-PCR)

MicroRNA qRT-PCR was carried out using Taqman probes as described by the manufacturer (Applied Biosystems, Warrington, UK) using 20 ng of microRNA per reaction in a Roche LightCycler 480 machine. Triplicate samples were used throughout. The mean Ct value of each triplicate was used for analysis, by the ΔC_t _method (ΔC_t _= mean C_t _of microRNA of interest-mean C_t _of U6). Expression levels were compared using Mann-Whitney independent *t-*test (Graphpad Prism v.4.0, La Jolla, CA).

### Survival analysis

Kaplan-Meier survival analysis was carried out on event free survival (EFS) times of MM patients as a function of microRNA expression, using the median value as cutoff. EFS was calculated as the time of diagnosis to the date of clinical relapse, death or last contact. Patients who were relapse-free at time of last contact were censored for analysis. Mean follow-up time was 20 months (range 1-60 months). Curves were compared by univariate (logrank) analysis using GraphPad Prism version 4.00 (La Jolla, CA).

## List of abbreviations

ANOVA: analysis of variance; *BCL2 *- B-cell CLL/lymphoma 2; *CCND1 *- cyclin D1; *DGCR8 *- DiGeorge syndrome critical region gene 8; EFS: event-free survival; *FGFR3 *- fibroblast growth factor receptor 3; FISH: fluorescent in situ hybridization; *HGMA1 *- Homogentisate 1,2-dioxygenase; IgH: immunoglobulin (heavy chain); *LMO2 *- LIM domain only 2 (rhombotin-like 1); *MCL1 *- myeloid cell leukemia sequence 1 (BCL2-related); MGUS: monoclonal gammopathy of undetermined significance; MIAME: Minimum Information About a Microarray Experiment; MM: multiple myeloma; PC: plasma cell; qRT-PCR: quantitative reverse transcriptase polymerase chain reaction; *RB1 *- retinoblastoma 1; SVM: support vector machine; SzS: Sezary syndrome;

## Competing interests

The authors declare that they have no competing interests.

## Authors' contributions

Conceived and designed the experiments: JC CHL CSRH TL JB JSW FP. Performed the experiments: JC EB X-HC DT CHL. Analyzed the data: ST X-HC CHL. Contributed reagents/materials/analysis tools: RK DH NJS. Wrote the paper: CHL JC. All authors read and approved the final manuscript.

## Reviewers' comments

Reviewer #1: Prof. Neil Smalheiser, Department of Psychiatry, University of Illinois, Chicago, IL, US.

### Reviewer's comments

This is a nice paper and I only have relatively minor comments, which can be addressed by page number:

p. 2. It would not hurt to have some discussion of the miR expression in the control group, in terms of the cell type profile, heterogeneity across individuals, etc. If you included that, I missed it.

#### Authors' response

*Whilst the reviewer has an interesting suggestion i.e. to look at variation in miRNA expression across the control group, the main theme of this research was to look at expression in multiple myeloma, therefore the number of controls samples used (n = 9) does not have sufficient power in order to address that specific question. However, our data does suggest that the control samples varied little in their miRNA expression profile as shown by unsupervised cluster analysis (*Figure 1*)*.

p. 2. You speak of altered miR expression in tumor cells as "aberrant" throughout the paper. Yet not every difference is necessarily aberrant. Some changes may be compensatory; some changes may simply reflect the physiological state of the tumor cells without being linked to tumorigenicity per se (e.g., they may differ in O2 tension or pH).

#### Authors' response

*We whole-heartedly agree that not all differentially expressed miRNAs identified in MM tumor cells will be directly linked with their neoplastic nature but instead may reflect indirect effects such as their abnormal microenvironment and/or interaction with non-tumor cells for example. We believe however that as these effects, direct and indirect, cumulatively represent tumor-associated changes in the miRNA expression profile of cells, that the use of the adjective 'aberrant' in this context is an accurate description of the expression behavior of miRNAs that are 'diverging from the normal' (Oxford Concise Dictionary)*.

p. 10 and Methods. It is worthwhile to describe the method of correcting p-values for multiple-testing in more detail.

#### Authors' response

*As stated in the methods section p-values were corrected using the Benjamini-Hogberg multiple testing correction algorithm as implemented in the ANOVA function of the Genespring software package. This algorithm is based on false discovery rate error type and further details can be found in the original publication of this method (Benjamini and Hochberg (1995). Journal of the Royal Statistical Society B, 57, 289-300)). As this method is widely used in microarray analyses we believe that a more in-depth and mathematical description of this method is not warranted for this manuscript*.

p. 10 and throughout. The paper has a nice balance between considering miRs as a clinical tool and as an indication of tumor biology. However, you can go still further to analyze in biological terms WHY the pattern of altered miR expression is the way it is, or (stated a different way) to analyze what dimensions are most important for SVM prediction. For example, do the altered miRs all share the same TFs driving them?

#### Authors' response

*The reviewer has raised a very important point about addressing the reason for aberrant miRNA expression in MM. However, the causal mechanisms behind miRNA dysregulation in any cancer are complex, although we did try to proffer some insight in the context of MM in both results and discussion sections of this manuscript. Possible mechanisms include chromosomal lesions at miRNA-encoding regions and defects in miRNA biosynthetic machinery, both of which were addressed in this manuscript. Further mechanisms include epigenetic regulation, changes in expression of genes such as E2F that can bind to promoter regions of pri-miRNA sequences, as well as any factor that can regulate levels of the protein-encoding genes that encode for >40% of human miRNAs. The problem with identifying genes such as TFs that are potentially targeted by commonly differentially expressed miRNAs, is that currently the predictive algorithms to carry out this type of analysis perform very poorly (Sethupathy, et al (2006) Nat Methods, **3**, 881-886). Therefore we believe that the robustness and hence usefulness of such an in silico approach is limited. Consequently we decided to restrict our discussion to those target genes that have been independently experimentally validated*.

p. 13. You mention that dicer and drosha tend to be up-regulated in B-cell malignancies. It would be desirable to have measured these in the SAME samples reported here and see if dicer and/or drosha levels are tightly correlated with miR up-regulation.

#### Authors' response

*This would be the ideal situation, and in the case of the DLBCL case this comparison was carried out (Lawrie et al (2009) Br. J. Haematol., **145**, 545-548). Unfortunately there was insufficient material left from the current study to carry out a similar analysis on the MM samples*.

p. 19, Methods. Were control and tumor samples collected during the same period? stored for similar amounts of time? Assayed in parallel? If not, this could create confounds.

#### Authors' response

*Due to the logistics of obtaining bone marrow samples from ~50 patients/controls it would have been extremely difficult to collect samples over the same period- samples were collected over a ~2 year period from two centers (Oxford and Zagreb). Patient/control samples were assayed in parallel and randomized/anonymized to the researchers processing them in order to minimize confounding variables*.

p. 20, Methods. Was U6 expression the same in control and tumor samples? This should be stated explicitly.

#### Authors' response

*U6 levels were measured in controls and tumor in parallel using the same assay system (i.e. Taqman probe qRT-PCR). There was no significant difference in absolute U6 expression levels (i.e. Ct values) between controls and tumor samples*.

p. 29, Table 1. You have mir-200a listed twice with two different values?! Please check this (very long) table.

#### Authors' response

*This was a mistake. Position 25 in *Table 1should have read miR-200a* and not miR-200a, this has been corrected.

p. 35, Figure 3 Legend. Please spell out DL, MM and SZ

#### Authors' response

*This issue has been addressed*.

Reviewer #2: Prof. Yuriy Gusev, Department of Surgery, University of Oklahoma, OK, USA.

This reviewer provided no comments for publication.

Reviewer #3: Unknown reviewer

### Reviewer's comments

This manuscript includes a description of microarray analysis for the expression of microRNA in purified CD138+ cells from 33 patients with multiple myeloma (MM), 5 patients with monoclonal gammopathy of undetermined significance (MGUS), and 9 healthy volunteers. The authors found distinct microRNA expression profiles from each group. Similar studies from 2 different groups have been reported. However, in one study only 16 samples from patients with MM, 6 from patients with MGUS, and 6 controls were included and in the another study samples from 52 patients but only 2 controls were studied. The manuscript is clear and concise. The data are convincing and the area of investigation is important. This manuscript is a valuable contribution. Specific comments follow:

1. The authors state several times that they have performed a comprehensive study to elucidate the complete miRNome of the CD138+ cells; however, I could not find how "complete" was defined. The manuscript would be improved by including a consideration about the completeness of the analysis performed. How the microarray analysis was defined as a complete analysis of miRNA and citations would enhance the manuscript.

#### Authors' response

*The 'complete miRNome' refers to the microarrays (μRNA microarrays) used for these experiments which contain probes against every known miRNA (the miRNome) as defined by the reference depository for miRNAs (miRBase- *http://www.mirbase.org*) (Griffiths-Jones et al (2006), Nucleic Acids Research, **34**, D140-144). At the time when these experiments were started (June 2009), the miRBase version was 13.0 which included 655 human sequences. The authors readily accept that the miRNA database has grown since this time and that the current version of the miRBase miRNome is version 16 and contains 1048 human sequences. This assertion has been modified in the text to read 'complete miRNome (miRBase version 13.0)' in the text in order to avoid confusion*.

2. The "leave-one-out cross-validation support vector machine algorithm" (mentioned on page 7) is not defined or described in the methods section. The support vector machine algorithm is also mentioned in the abstract but the manuscript would be improved by describing and referencing this technique.

#### Authors' response

*The support vector machine (SVM) algorithm is a supervised learning method commonly used for classification in microarray experiments (along with k-nearest neighbor algorithm) and is based on non-probabilistic binary linear classification (Cortes & Vapnik (1995) Machine learning, **20**). We believe that as the parameters used for its implementation within the Genespring package as described in the materials and methods section are already given, that this would be sufficient information for others to carry out the same analysis*.

3. In the results the authors state that 8 miRNA were chosen to be measured by qRT-PCR to validate the microarray data. They state, "These data were consistent with the microarray results". In the discussion the authors indicate that "miR-32 and miR-181a were identified as being up-regulated by microarray analysis" by qRT-PCR failed to show any significant differences in expression levels. Were there other miRNA studied by qRT-PCR? Why weren't miR-32 and miR181a included in Figure 2?

#### Authors' response

*The eight miRNAs that were tested by qRT-PCR shown in *Figure 2*were chosen on the basis that they were differentially expressed in our microarray analysis and had previously been shown to be dysregulated in MM or other hematological malignancies. As all of these miRNAs were also significantly differentially expressed when measured by qRT-PCR they were described as being 'consistent with the microarray data'. The only other miRNAs that we measured (miR-32 and miR-181a) by qRT-PCR which were also chosen on the basis shown to be dysregulated in previous studies but we did not find them to be significantly different by qRT-PCR which is why the data was not included in *Figure 2.

4. In the discussion the authors state that the investigators of reference 17 "only examined 10 MM and 4 controls". In the abstract of this manuscript, Pichiorri et al. indicate that 16 samples from patients with MM were studied along with 6 with MGUS and 6 normal donors. Also, in this same spot of the discussion, the authors indicate that the investigators of reference 16 only studied 2 controls but the 52 patient samples analyzed was not mentioned. Moreover, the purpose of that study was to risk stratification.

#### Authors' response

*These comments in the discussion were made in the context of the identification of aberrantly expressed miRNA associated with MM diagnosis. The Pichiorri study states in the results section (pg 12886) that their MM-associated miRNA signature was derived from the array data of 41 MM cell lines, 10 MM patients and 4 controls. Whereas for the Zhou et al study (pg 7904) the MM miRNA signature was derived from a comparison of array data from 52 MM cases with samples from 2 healthy donors. Whilst it is true that the Zhou et al study mainly focused on the identification of risk groups within the 52 MM cases, a significant part of the results section of this paper refers to the identity of miRNAs that are differentially expressed between controls and MM cases. The point that we were making in the discussion is that insufficient numbers of controls were used in both of these previous studies, and that our study was therefore an improvement on these studies, in the context of the identification of aberrantly expressed miRNAs in MM tumor cells*.

## Acknowledgements and Funding

This work was funded by grants from Leukaemia and Lymphoma Research (JC, EB, X-HC, DT, JB and JSW) and the Julian Starmer-Smith Memorial Fund (CHL). The authors acknowledge financial support from the Department of Health via the National Institute for Health Research (NIHR) comprehensive Biomedical Research Centre award to Oxford Radcliffe NHS Trust.

The authors wish to thank Maite Cabes, Graham Collins, Narendra Kaushik, Andy Campbell and Pamela Roberts (John Radcliffe Hospital) for their help in collecting patient material and collating clinical data. We are indebted to Fiona Ross and her colleagues at the LLR Myeloma Cytogenetic Database, Wessex Regional Genetic Laboratory, Salisbury, UK for their help in implementing and interpreting the microFISH technique as well as their input in the writing this manuscript.

## Supplementary Material

Additional file 1 **Additional tables (Tables S1-S7) and figure (Figures S1-S4)**. **Table S1**. Clinical details of MM and MGUS patients. **Table S2**. Table of microRNAs aberrantly expressed in DLBCL, SzS and MM, highlighting those that are common. **Table S3**. MicroRNAs differentially expressed (*P *< 0.05) between MGUS (n = 5) and controls (n = 9). **Table S4**. MicroRNAs differentially expressed (*P *< 0.05) between MM (n = 32) and MGUS (n = 5). **Table S5**. MicroRNAs differentially expressed (*P *< 0.05) between IgA (n = 8) and IgG (n = 13) isotype MM cases. **Table S6**. MicroRNAs differentially expressed (*P *< 0.05) between LC-only myeloma (n = 8) and non-LC-only myeloma MM cases (n = 21). **Table S7**. MicroRNAs associated with event-free survival (EFS) in MM cases. **Figure S1**. Heat map depicting cluster analysis of MM and control samples on the basis of expression values of 129 MM-associated microRNAs. **Figure S2**. Heat map depicting cluster analysis of MGUS and control samples on the basis of expression values of 39 MGUS-associated microRNAs. **Figure S3**. Venn-diagram depicting relationship between microRNAs differentially expressed in MGUS, MM and controls. **Figure S4**. Heat map depicting cluster analysis of IgG and IgA isotype MM cases on the basis of expression values of 21 isotype-associated microRNAs (Table S5). click here for file

## References

[B1] Ries L Eisner M Kosary C SEER Cancer Statistics Review, 1975-2001 2004 Bethesda: National Cancer Institute

[B2] Mateos MV Richardson PG Schlag R Khuageva NK Dimopoulos MA Shpilberg O Kropff M Spicka I Petrucci MT Palumbo A Bortezomib plus melphalan and prednisone compared with melphalan and prednisone in previously untreated multiple myeloma: updated follow-up and impact of subsequent therapy in the phase III VISTA trial J Clin Oncol 2010 28 2259 2266 10.1200/JCO.2009.26.0638 20368561

[B3] Calin GA Ferracin M Cimmino A Di Leva G Shimizu M Wojcik SE Iorio MV Visone R Sever NI Fabbri M A MicroRNA signature associated with prognosis and progression in chronic lymphocytic leukemia N Engl J Med 2005 353 1793 1801 10.1056/NEJMoa050995 16251535

[B4] Iorio MV Ferracin M Liu CG Veronese A Spizzo R Sabbioni S Magri E Pedriali M Fabbri M Campiglio M MicroRNA gene expression deregulation in human breast cancer Cancer Res 2005 65 7065 7070 10.1158/0008-5472.CAN-05-1783 16103053

[B5] Lawrie CH Soneji S Marafioti T Cooper CD Palazzo S Paterson JC Cattan H Enver T Mager R Boultwood J MicroRNA expression distinguishes between germinal center B cell-like and activated B cell-like subtypes of diffuse large B cell lymphoma Int J Cancer 2007 121 1156 1161 10.1002/ijc.22800 17487835

[B6] Lawrie CH Gal S Dunlop HM Pushkaran B Liggins AP Pulford K Banham AH Pezzella F Boultwood J Wainscoat JS Detection of elevated levels of tumour-associated microRNAs in serum of patients with diffuse large B-cell lymphoma Br J Haematol 2008 141 672 675 10.1111/j.1365-2141.2008.07077.x 18318758

[B7] Lawrie CH Chi J Taylor S Tramonti D Ballabio E Palazzo S Saunders NJ Pezzella F Boultwood J Wainscoat JS Hatton CS Expression of microRNAs in diffuse large B cell lymphoma is associated with immunophenotype, survival and transformation from follicular lymphoma J Cell Mol Med 2009 13 1248 1260 10.1111/j.1582-4934.2008.00628.x 19413891 PMC4496139

[B8] Lu J Getz G Miska EA Alvarez-Saavedra E Lamb J Peck D Sweet-Cordero A Ebert BL Mak RH Ferrando AA MicroRNA expression profiles classify human cancers Nature 2005 435 834 838 10.1038/nature03702 15944708

[B9] Lawrie CH microRNA expression in lymphoid malignancies: new hope for diagnosis and therapy? J Cell Mol Med 2008 12 1432 1444 10.1111/j.1582-4934.2008.00399.x 18624758 PMC3918059

[B10] Ballabio E Mitchell T van Kester MS Taylor S Dunlop HM Chi J Tosi I Vermeer MH Tramonti D Saunders NJ MicroRNA expression in Sezary syndrome: identification, function, and diagnostic potential Blood 2010 116 1105 1113 10.1182/blood-2009-12-256719 20448109 PMC2938132

[B11] Zhou Y Chen L Barlogie B Stephens O Wu X Williams DR Cartron MA van Rhee F Nair B Waheed S High-risk myeloma is associated with global elevation of miRNAs and overexpression of EIF2C2/AGO2 Proc Natl Acad Sci USA 107 7904 7909 10.1073/pnas.0908441107 PMC2867889 20385818

[B12] Pichiorri F Suh SS Ladetto M Kuehl M Palumbo T Drandi D Taccioli C Zanesi N Alder H Hagan JP MicroRNAs regulate critical genes associated with multiple myeloma pathogenesis Proc Natl Acad Sci USA 2008 105 12885 12890 10.1073/pnas.0806202105 18728182 PMC2529070

[B13] Lee Y Jeon K Lee JT Kim S Kim VN MicroRNA maturation: stepwise processing and subcellular localization EMBO J 2002 21 4663 4670 10.1093/emboj/cdf476 12198168 PMC126204

[B14] Kyle RA Therneau TM Rajkumar SV Larson DR Plevak MF Offord JR Dispenzieri A Katzmann JA Melton LJ Prevalence of monoclonal gammopathy of undetermined significance N Engl J Med 2006 354 3 1362 1369 10.1056/NEJMoa054494 16571879

[B15] Zhan F Huang Y Colla S Stewart JP Hanamura I Gupta S Epstein J Yaccoby S Sawyer J Burington B The molecular classification of multiple myeloma Blood 2006 108 2020 2028 10.1182/blood-2005-11-013458 16728703 PMC1895543

[B16] Fonseca R Bergsagel PL Drach J Shaughnessy J Gutierrez N Stewart AK Morgan G Van Ness B Chesi M Minvielle S International Myeloma Working Group molecular classification of multiple myeloma: spotlight review Leukemia 2009 23 2210 2221 10.1038/leu.2009.174 19798094 PMC2964268

[B17] Ross FM Ibrahim AH Vilain-Holmes A Winfield MO Chiecchio L Protheroe RK Strike P Gunasekera JL Jones A Harrison CJ Age has a profound effect on the incidence and significance of chromosome abnormalities in myeloma Leukemia 2005 19 1634 1642 10.1038/sj.leu.2403857 15990862

[B18] Kuehl WM Bergsagel PL Early genetic events provide the basis for a clinical classification of multiple myeloma Hematology (Am Soc Hematol Educ Program) 2005 346 352 10.1182/asheducation-2005.1.346 16304402

[B19] Gutierrez NC Sarasquete ME Misiewicz-Krzeminska I Delgado M De Las Rivas J Ticona FV Ferminan E Martin-Jimenez P Chillon C Risueno A Deregulation of microRNA expression in the different genetic subtypes of multiple myeloma and correlation with gene expression profiling Leukemia 2010 24 629 637 10.1038/leu.2009.274 20054351

[B20] Lionetti M Biasiolo M Agnelli L Todoerti K Mosca L Fabris S Sales G Deliliers GL Bicciato S Lombardi L Identification of microRNA expression patterns and definition of a microRNA/mRNA regulatory network in distinct molecular groups of multiple myeloma Blood 2009 114 e20 26 10.1182/blood-2009-08-237495 19846888

[B21] Greipp PR San Miguel J Durie BG Crowley JJ Barlogie B Blade J Boccadoro M Child JA Avet-Loiseau H Kyle RA International staging system for multiple myeloma J Clin Oncol 2005 23 3412 3420 10.1200/JCO.2005.04.242 15809451

[B22] Sirohi B Powles R Kulkarni S Rudin C Saso R Lal R Singhal S Mehta J Horton C Treleaven J Comparison of new patients with Bence-Jones, IgG and IgA myeloma receiving sequential therapy: the need to regard these immunologic subtypes as separate disease entities with specific prognostic criteria Bone Marrow Transplant 2001 28 29 37 10.1038/sj.bmt.1703093 11498741

[B23] Kyle RA Gertz MA Witzig TE Lust JA Lacy MQ Dispenzieri A Fonseca R Rajkumar SV Offord JR Larson DR Review of 1027 patients with newly diagnosed multiple myeloma Mayo Clin Proc 2003 78 21 33 10.4065/78.1.21 12528874

[B24] Shustik C Bergsagel DE Pruzanski W Kappa and lambda light chain disease: survival rates and clinical manifestations Blood 1976 48 41 51 820387

[B25] Huang Q Gumireddy K Schrier M le Sage C Nagel R Nair S Egan DA Li A Huang G Klein-Szanto AJ The microRNAs miR-373 and miR-520c promote tumour invasion and metastasis Nat Cell Biol 2008 10 202 210 10.1038/ncb1681 18193036

[B26] Lawrie CH Saunders NJ Soneji S Palazzo S Dunlop HM Cooper CD Brown PJ Troussard X Mossafa H Enver T MicroRNA expression in lymphocyte development and malignancy Leukemia 2008 22 1440 1446 10.1038/sj.leu.2405083 18185523

[B27] Zhang J Jima DD Jacobs C Fischer R Gottwein E Huang G Lugar PL Lagoo AS Rizzieri DA Friedman DR Patterns of microRNA expression characterize stages of human B cell differentiation Blood 2009 113 4586 4594 10.1182/blood-2008-09-178186 19202128 PMC2680365

[B28] Merkel O Hamacher F Laimer D Sifft E Trajanoski Z Scheideler M Egger G Hassler MR Thallinger C Schmatz A Identification of differential and functionally active miRNAs in both anaplastic lymphoma kinase (ALK)+ and ALK- anaplastic large-cell lymphoma Proc Natl Acad Sci USA 2010 107 16228 16233 10.1073/pnas.1009719107 20805506 PMC2941277

[B29] Lawrie CH Cooper CD Ballabio E Chi J Tramonti D Hatton CS Aberrant expression of microRNA biosynthetic pathway components is a common feature of haematological malignancy Br J Haematol 2009 145 545 548 10.1111/j.1365-2141.2009.07642.x 19298586

[B30] Wong QW Lung RW Law PT Lai PB Chan KY To KF Wong N MicroRNA-223 is commonly repressed in hepatocellular carcinoma and potentiates expression of Stathmin1 Gastroenterology 2008 135 257 269 10.1053/j.gastro.2008.04.003 18555017

[B31] Fazi F Racanicchi S Zardo G Starnes LM Mancini M Travaglini L Diverio D Ammatuna E Cimino G Lo-Coco F Epigenetic silencing of the myelopoiesis regulator microRNA-223 by the AML1/ETO oncoprotein Cancer Cell 2007 12 457 466 10.1016/j.ccr.2007.09.020 17996649

[B32] Stamatopoulos B Meuleman N Haibe-Kains B Saussoy P Van Den Neste E Michaux L Heimann P Martiat P Bron D Lagneaux L microRNA-29c and microRNA-223 down-regulation has in vivo significance in chronic lymphocytic leukemia and improves disease risk stratification Blood 2009 113 5237 5245 10.1182/blood-2008-11-189407 19144983

[B33] Lawrie CH Ballabio E Dyar OJ Jones M Ventura R Chi J Tramonti D Gooding S Boultwood J Wainscoat JS MicroRNA expression in chronic lymphocytic leukaemia Br J Haematol 2009 147 398 402 10.1111/j.1365-2141.2009.07857.x 19681887

[B34] Felli N Pedini F Romania P Biffoni M Morsilli O Castelli G Santoro S Chicarella S Sorrentino A Peschle C Marziali G MicroRNA 223-dependent expression of LMO2 regulates normal erythropoiesis Haematologica 2009 94 479 486 10.3324/haematol.2008.002345 19278969 PMC2663611

[B35] Natkunam Y Zhao S Mason DY Chen J Taidi B Jones M Hammer AS Hamilton Dutoit S Lossos IS Levy R The oncoprotein LMO2 is expressed in normal germinal-center B cells and in human B-cell lymphomas Blood 2007 109 1636 1642 10.1182/blood-2006-08-039024 17038524 PMC1794056

[B36] Akao Y Nakagawa Y Kitade Y Kinoshita T Naoe T Downregulation of microRNAs-143 and -145 in B-cell malignancies Cancer Sci 2007 98 1914 1920 10.1111/j.1349-7006.2007.00618.x 17892514 PMC11158757

[B37] Landgren O Kyle RA Pfeiffer RM Katzmann JA Caporaso NE Hayes RB Dispenzieri A Kumar S Clark RJ Baris D Monoclonal gammopathy of undetermined significance (MGUS) consistently precedes multiple myeloma: a prospective study Blood 2009 113 5412 5417 10.1182/blood-2008-12-194241 19179464 PMC2689042

[B38] Chan JA Krichevsky AM Kosik KS MicroRNA-21 is an antiapoptotic factor in human glioblastoma cells Cancer Res 2005 65 6029 6033 10.1158/0008-5472.CAN-05-0137 16024602

[B39] Si ML Zhu S Wu H Lu Z Wu F Mo YY miR-21-mediated tumor growth Oncogene 2006 26 2799 2803 17072344 10.1038/sj.onc.1210083

[B40] Fonseca R Oken MM Harrington D Bailey RJ Van Wier SA Henderson KJ Kay NE Van Ness B Greipp PR Dewald GW Deletions of chromosome 13 in multiple myeloma identified by interphase FISH usually denote large deletions of the q arm or monosomy Leukemia 2001 15 981 986 10.1038/sj.leu.2402125 11417487

[B41] He L Thomson JM Hemann MT Hernando-Monge E Mu D Goodson S Powers S Cordon-Cardo C Lowe SW Hannon GJ Hammond SM A microRNA polycistron as a potential human oncogene Nature 2005 435 828 833 10.1038/nature03552 15944707 PMC4599349

[B42] Zojer N Konigsberg R Ackermann J Fritz E Dallinger S Kromer E Kaufmann H Riedl L Gisslinger H Schreiber S Deletion of 13q14 remains an independent adverse prognostic variable in multiple myeloma despite its frequent detection by interphase fluorescence in situ hybridization Blood 2000 95 1925 1930 10706856

[B43] Lawrie CH MicroRNAs and haematology: small molecules, big function Br J Haematol 2007 137 503 512 10.1111/j.1365-2141.2007.06611.x 17539773

[B44] Cloonan N Brown MK Steptoe AL Wani S Chan WL Forrest AR Kolle G Gabrielli B Grimmond SM The miR-17-5p microRNA is a key regulator of the G1/S phase cell cycle transition Genome Biol 2008 9 R127 10.1186/gb-2008-9-8-r127 18700987 PMC2575517

[B45] Aguda BD Kim Y Piper-Hunter MG Friedman A Marsh CB MicroRNA regulation of a cancer network: consequences of the feedback loops involving miR-17-92, E2F, and Myc Proc Natl Acad Sci USA 2008 105 19678 19683 10.1073/pnas.0811166106 19066217 PMC2598727

[B46] Hossain A Kuo MT Saunders GF Mir-17-5p regulates breast cancer cell proliferation by inhibiting translation of AIB1 mRNA Mol Cell Biol 2006 26 8191 8201 10.1128/MCB.00242-06 16940181 PMC1636750

[B47] Xu J Liao X Wong C Downregulations of B-cell lymphoma 2 and myeloid cell leukemia sequence 1 by microRNA 153 induce apoptosis in a glioblastoma cell line DBTRG-05MG Int J Cancer 2010 126 1029 1035 19676043 10.1002/ijc.24823

[B48] Wuilleme-Toumi S Robillard N Gomez P Moreau P Le Gouill S Avet-Loiseau H Harousseau JL Amiot M Bataille R Mcl-1 is overexpressed in multiple myeloma and associated with relapse and shorter survival Leukemia 2005 19 1248 1252 10.1038/sj.leu.2403784 15902294

[B49] Hiroki E Akahira J Suzuki F Nagase S Ito K Suzuki T Sasano H Yaegashi N Changes in microRNA expression levels correlate with clinicopathological features and prognoses in endometrial serous adenocarcinomas Cancer Sci 2010 101 241 249 10.1111/j.1349-7006.2009.01385.x 19891660 PMC11159282

[B50] Wei JJ Wu X Peng Y Shi G Olca B Yang X Daniels G Osman I Ouyang J Hernando E Regulation of HMGA1 expression by microRNA296 affects prostate cancer growth and invasion Clin Cancer Res 2010 10.1158/1078-0432.CCR-20-0229 32122931

[B51] Durie BG Kyle RA Belch A Bensinger W Blade J Boccadoro M Child JA Comenzo R Djulbegovic B Fantl D Myeloma management guidelines: a consensus report from the Scientific Advisors of the International Myeloma Foundation Hematol J 2003 4 379 398 10.1038/sj.thj.6200312 14671610

[B52] Wuilleme S Robillard N Lode L Magrangeas F Beris H Harousseau JL Proffitt J Minvielle S Avet-Loiseau H Ploidy, as detected by fluorescence in situ hybridization, defines different subgroups in multiple myeloma Leukemia 2005 19 275 278 10.1038/sj.leu.2403586 15538401

[B53] Smyth GK Speed T Normalization of cDNA microarray data Methods 2003 31 265 273 10.1016/S1046-2023(03)00155-5 14597310

