# Supplementary material for: MicroRNA expression in multiple myeloma is associated with genetic subtype, isotype and survival
Source: Biol Direct. 2011 May 18;6:23. doi: 10.1186/1745-6150-6-23 (PMC3120802; doi:10.1186/1745-6150-6-23)
Supplement: Additional file 1 — Additional tables (Tables S1-S7) and figure (Figures S1-S4). Table S1. Clinical details of MM and MGUS patients. Table S2. Table of microRNAs aberrantly expressed in DLBCL, SzS and MM, highlighting those that are common. Table S3. MicroRNAs differentially expressed (P < 0.05) between MGUS (n = 5) and controls (n = 9). Table S4. MicroRNAs differentially expressed (P < 0.05) between MM (n = 32) and MGUS (n = 5). Table S5. MicroRNAs differentially expressed (P < 0.05) between IgA (n = 8) and IgG (n = 13) isotype MM cases. Table S6. MicroRNAs differentially expressed (P < 0.05) between LC-only myeloma (n = 8) and non-LC-only myeloma MM cases (n = 21). Table S7. MicroRNAs associated with event-free survival (EFS) in MM cases. Figure S1. Heat map depicting cluster analysis of MM and control samples on the basis of expression values of 129 MM-associated microRNAs. Figure S2. Heat map depicting cluster analysis of MGUS and control samples on the basis of expression values of 39 MGUS-associated microRNAs. Figure S3. Venn-diagram depicting relationship between microRNAs differentially expressed in MGUS, MM and controls. Figure S4. Heat map depicting cluster analysis of IgG and IgA isotype MM cases on the basis of expression values of 21 isotype-associated microRNAs (Table S5). [file 1745-6150-6-23-S1.DOC]

**Table S1.** Clinical details of MM and MGUS patients used in analysis depicting isotype, karyotype and International Staging System (ISS) score [1].

| **ID** | **Age** | **Sex** | **Isotype** | **Karyotype** | **ISS** |
| --- | --- | --- | --- | --- | --- |
| MGUS#1 | 50 | M | IgG | NK | NK |
| MGUS#2 | 67 | M | IgG | NK | NK |
| MGUS#4 | 86 | M | IgG | NK | NK |
| MGUS#5 | 71 | F | IgG | NK | NK |
| MGUS#6 | 73 | M | IgG | NK | NK |
| MM#7 | 71 | M | IgG | t(11;14) | 3 |
| MM#8 | 63 | M | LC | NDA | 2 |
| MM#9 | 81 | F | IgG | t(14;?) | 2 |
| MM#10 | 60 | F | LC | NDA | 3 |
| MM#11 | 66 | F | IgG | del(13q) | 1 |
| MM#12 | 67 | M | IgG | NDA | 1 |
| MM#13 | 69 | M | IgA | t(14;?) | 3 |
| MM#14 | 47 | M | IgA | t(4;14)* | 2 |
| MM#15 | 89 | M | IgG | NDA | 1 |
| MM#16 | 62 | F | IgA | NDA | 2 |
| MM#18 | 62 | M | LC | t(4;14)* | 3 |
| MM#19 | 88 | M | LC | NDA | 3 |
| MM#21 | 85 | M | IgG | t(14;?) | 1 |
| MM#23 | 55 | F | IgG | NDA | 1 |
| MM#24 | 61 | M | IgG | t(11;14), del(13q) | 2 |
| MM#25 | 86 | F | IgA | t(4;14), del(13q) | 3 |
| MM#26 | 56 | M | IgG | NK | 2 |
| MM#27 | 58 | M | LC | NDA | 2 |
| MM#28 | 76 | F | IgG | NK | 3 |
| MM#29 | 78 | F | IgG | NK | 2 |
| MM#30 | 67 | M | LC | NK | 3 |
| MM#31 | 72 | F | IgA | t(11;14), del(13q) | 2 |
| MM#32 | 51 | F | IgG | t(11;14) | 3 |
| MM#33 | 88 | F | IgG | t(11;14), del(13q) | NK |
| MM#34 | 60 | M | NK | t(11;14), del(13q) | NK |
| MM#35 | 61 | M | LC | NDA | NK |
| MM#36 | 65 | M | LC | t(11;14) | 3 |
| MM#41 | 63 | M | IgA | del(13q) | 1 |
| MM#43 | 73 | F | IgA | NK | 1 |
| MM#44 | 62 | M | IgG | NK | NK |
| MM#45 | 43 | M | NK | NDA | NK |
| MM#46 | 60 | M | IgA | NK | NK |
| MM#47 | 51 | F | NK | t(11;14) | NK |

**Notes:** NK, not known; LC, light chain only; HPD, hyperdiploidy; t(11;14), IgH;CCND1translocation; t(4;14), IgH;FGFR3 translocation; t(14;?), IgH translocation partner unknown.* insufficient material available to test del(13q) probe. NDA, no detectable abnormality.

**Table S2.** Table of microRNAs aberrantly expressed in DLBCL [2], SzS [3] and MM (Table 1), showing commonly expressed microRNAs.

| **DLBCL** | **MM** | **SzS** |
| --- | --- | --- |
| ***miR-100*** | *miR-1* | *let-7g* |
| *miR-10a* | ***miR-100*** | *let-7i* |
| *miR-10b* | *miR-106b* | ***miR-100*** |
| *miR-125a* | *miR-1227* | *miR-103* |
| *miR-125b* | *miR-1234* | *miR-106a* |
| *miR-126* | *miR-125b* | *miR-106b* |
| *miR-126** | ***miR-128a*** | *miR-107* |
| ***miR-128a*** | ***miR-128b*** | *miR-10a* |
| ***miR-128b*** | *miR-129-3p* | *miR-125a* |
| ***miR-133b*** | *miR-130a* | *miR-125b* |
| *miR-135a* | *miR-130b* | ***miR-128a*** |
| *miR-135b* | *miR-133a* | ***miR-128b*** |
| *miR-136* | ***miR-133b*** | ***miR-133b*** |
| *miR-138* | *miR-135a* | *miR-140-3p* |
| ***miR-143*** | *miR-135b* | *miR-140-5p* |
| *miR-145* | *miR-140* | *miR-141* |
| *miR-150* | *miR-140-3p* | *miR-142-3p* |
| *miR-155* | *miR-140-5p* | *miR-142-5p* |
| ***miR-181a*** | *miR-141* | ***miR-143*** |
| *miR-181b* | *miR-142-5p* | *miR-145* |
| *miR-181c* | ***miR-143*** | *miR-146a* |
| *miR-181d* | *miR-146b* | *miR-146b* |
| *miR-182* | *miR-151* | *miR-148a* |
| *miR-183* | *miR-151-3p* | *miR-148b* |
| *miR-189* | ***miR-181a*** | *miR-150* |
| *miR-195* | *miR-182* | *miR-151* |
| *miR-199a* | *miR-183* | *miR-152* |
| *miR-199a** | *miR-188* | *miR-15a* |
| *miR-199b* | *miR-188-5p* | *miR-15b* |
| *miR-205* | *miR-18a* | *miR-16* |
| *miR-206* | *miR-190b* | *miR-17* |
| *miR-21* | *miR-191** | *miR-17-5p* |
| *miR-214* | *miR-192* | ***miR-181a*** |
| *miR-218* | *miR-194* | *miR-181b* |
| ***miR-223*** | *miR-19a* | *miR-181c* |
| *miR-26a* | *miR-19b* | *miR-185* |
| *miR-26b* | *miR-200a* | *miR-186* |
| ***miR-335*** | *miR-200b* | *miR-189* |
| *miR-34a* | *miR-200c* | *miR-18a* |
| ***miR-361*** | *miR-202* | *miR-191* |
| *miR-363* | *miR-203* | *miR-192* |
| *miR-365* | *miR-205* | *miR-194* |
| *miR-451* | *miR-206* | *miR-199a** |
| ***miR-455*** | *miR-20b* | *miR-19a* |
| *miR-495* | *miR-210* | *miR-19b* |
| *miR-497* | *miR-212* | *miR-200c* |
| *miR-550* | *miR-218* | *miR-20a* |
| ***miR-574*** | *miR-220* | *miR-20b* |
| *miR-584* | ***miR-223*** | *miR-214* |
| *miR-594* | *miR-27a* | *miR-215* |
| *miR-625* | *miR-27b* | *miR-22* |
| *miR-638* | *miR-28* | ***miR-223*** |
| ***miR-652*** | *miR-28-3p* | *miR-23a* |
| *miR-766* | *miR-300* | *miR-23b* |
| *miR-768-5p* | *miR-30e-5p* | *miR-24* |
| *miR-801* | *miR-31* | *miR-25* |
| *miR-9* | *miR-32* | *miR-26a* |
| *miR-9** | *miR-320* | *miR-26b* |
| ***miR-99a*** | ***miR-335*** | *miR-27a* |
| *miR-99b* | *miR-337* | *miR-27b* |
|  | *miR-338* | *miR-28* |
|  | *miR-338-5p* | *miR-28-3p* |
|  | *miR-340* | *miR-29a* |
|  | *miR-342* | *miR-29b* |
|  | *miR-34a* | *miR-29c* |
|  | *miR-34b* | *miR-30a-3p* |
|  | *miR-34c-3p* | *miR-30a-5p* |
|  | ***miR-361*** | *miR-30b* |
|  | *miR-369-3p* | *miR-30c* |
|  | *miR-371-3p* | *miR-30d* |
|  | *miR-373** | *miR-30e* |
|  | *miR-378* | *miR-30e-5p* |
|  | *miR-378** | *miR-31* |
|  | *miR-383* | *miR-32* |
|  | *miR-412* | *miR-320* |
|  | *miR-421* | ***miR-335*** |
|  | *miR-422b* | *miR-338* |
|  | *miR-432* | *miR-338-3p* |
|  | *miR-452* | *miR-340* |
|  | *miR-454* | *miR-342* |
|  | ***miR-455*** | *miR-345* |
|  | *miR-485-5p* | ***miR-361*** |
|  | *miR-491-5p* | *miR-361-3p* |
|  | *miR-499-5p* | *miR-362-3p* |
|  | *miR-502* | *miR-363* |
|  | *miR-515-3p* | *miR-365* |
|  | *miR-516-3p* | *miR-374* |
|  | *miR-518d* | *miR-374b* |
|  | *miR-519d* | *miR-376a* |
|  | *miR-520b* | *miR-378* |
|  | *miR-520g* | *miR-422b* |
|  | *miR-520h* | *miR-423* |
|  | *miR-524** | *miR-423-3p* |
|  | *miR-532-3p* | *miR-425-5p* |
|  | *miR-548d-5p* | *miR-429* |
|  | *miR-549* | ***miR-455*** |
|  | ***miR-574*** | *miR-484* |
|  | *miR-576-3p* | *miR-500* |
|  | *miR-589* | *miR-505* |
|  | *miR-590* | *miR-518a* |
|  | *miR-590-3p* | *miR-518d* |
|  | *miR-593* | *miR-532* |
|  | *miR-603* | *miR-532-3p* |
|  | *miR-608* | *miR-565* |
|  | *miR-610* | ***miR-574*** |
|  | *miR-616* | *miR-582* |
|  | *miR-621* | *miR-590* |
|  | *miR-624* | *miR-590-3p* |
|  | *miR-625* | *miR-594* |
|  | *miR-628-5p* | ***miR-652*** |
|  | *miR-629* | *miR-660* |
|  | *miR-631* | *miR-7* |
|  | *miR-634* | *miR-768-5p* |
|  | *miR-637* | *miR-801* |
|  | *miR-649* | *miR-92* |
|  | ***miR-652*** | *miR-92a* |
|  | *miR-660* | *miR-92b* |
|  | *miR-671-3p* | *miR-93* |
|  | *miR-7* | *miR-95* |
|  | *miR-708* | *miR-98* |
|  | *miR-760* | ***miR-99a*** |
|  | *miR-802* |  |
|  | *miR-877* |  |
|  | *miR-885-3p* |  |
|  | *miR-885-5p* |  |
|  | *miR-891a* |  |
|  | *miR-9* |  |
|  | *miR-9** |  |
|  | *miR-98* |  |
|  | ***miR-99a*** |  |

**Notes.** *miR-x*, aberrantly expressed in DLBCL and SzS; *miR-x*, aberrantly expressed in MM and SzS; *miR-x*, aberrantly expressed in DL and MM; ***miR-x***, aberrantly expressed in MM, DL and SzS.

**Table S3.** MicroRNAs differentially expressed (*P* < 0.05) between MGUS (n = 5) and controls (n = 9). MicroRNAs also identified by Pichiorri *et al* [4] are indicated in bold type. MicroRNAs also aberrantly expressed in MM (Table 1) are depicted in red font.

| **microRNA** | **Fold change** | ***P*-value** |
| --- | --- | --- |
| miR-593 | 3.56 | 8.17E-04 |
| miR-342 | 5.31 | 8.31E-04 |
| miR-454 | 3.74 | 1.27E-03 |
| **miR-21** | 10.33 | 1.49E-03 |
| miR-509 | 2.31 | 2.32E-03 |
| **miR-210** | 4.56 | 3.03E-03 |
| **miR-9*** | 3.24 | 4.46E-03 |
| miR-760 | 2.41 | 5.23E-03 |
| miR-891a | 2.98 | 6.26E-03 |
| miR-155 | 8.10 | 6.64E-03 |
| **miR-200b** | 2.51 | 8.13E-03 |
| miR-34b | 2.62 | 1.11E-02 |
| miR-453 | 2.96 | 1.28E-02 |
| miR-412 | 2.10 | 1.54E-02 |
| miR-143 | 5.89 | 2.01E-02 |
| miR-220 | 1.69 | 2.16E-02 |
| **miR-222** | 1.90 | 2.25E-02 |
| miR-206 | 2.32 | 2.50E-02 |
| miR-632 | 1.75 | 2.55E-02 |
| miR-188-5p | 2.17 | 2.57E-02 |
| miR-885-3p | 2.34 | 2.70E-02 |
| **miR-376** | 1.89 | 2.74E-02 |
| miR-654-3p | 2.66 | 3.09E-02 |
| miR-625 | 2.56 | 3.15E-02 |
| miR-629 | 1.95 | 3.55E-02 |
| miR-616 | 1.89 | 4.23E-02 |
| miR-601 | 2.12 | 4.73E-02 |
| miR-432 | 2.90 | 4.78E-02 |
| miR-548b | -2.89 | 2.50E-03 |
| miR-216 | -2.19 | 5.99E-03 |
| miR-373* | -2.14 | 1.08E-02 |
| miR-296 | -1.70 | 1.44E-02 |
| **miR-339** | -1.69 | 2.16E-02 |
| miR-208 | -1.78 | 2.40E-02 |
| miR-380-5p | -1.78 | 2.69E-02 |
| miR-381 | -1.47 | 2.89E-02 |
| miR-518c | -1.95 | 3.73E-02 |
| miR-519d | -2.05 | 4.27E-02 |
| **miR-328** | -1.55 | 4.65E-02 |

**Table S4.** MicroRNAs differentially expressed (*P* < 0.05) between MM (n = 32) and MGUS (n = 5). MicroRNAs also associated with MGUS vs. control are depicted in red font. Positive values are up-regulated in MM.

| **microRNA** | **Fold change** | ***P*-value** |
| --- | --- | --- |
| miR-548b | 2.40 | 4.65E-05 |
| miR-455-3p | 2.41 | 9.40E-04 |
| miR-362 | 1.79 | 1.20E-03 |
| miR-502 | 2.01 | 1.63E-03 |
| miR-188 | 2.08 | 2.54E-03 |
| miR-515-3p | 4.92 | 3.45E-03 |
| miR-302c* | 1.70 | 6.90E-03 |
| miR-140 | 2.35 | 7.79E-03 |
| miR-500 | 3.37 | 7.80E-03 |
| miR-490 | 1.92 | 8.61E-03 |
| miR-329 | 2.37 | 9.54E-03 |
| miR-345 | 1.36 | 1.06E-02 |
| miR-362-3p | 2.51 | 1.08E-02 |
| miR-300 | 2.56 | 1.22E-02 |
| miR-301 | 2.43 | 1.43E-02 |
| miR-151 | 2.21 | 1.46E-02 |
| miR-192 | 1.93 | 1.53E-02 |
| miR-339-5p | 1.54 | 1.74E-02 |
| miR-381 | 1.50 | 2.04E-02 |
| miR-34c | 1.44 | 2.06E-02 |
| miR-340 | 2.35 | 2.63E-02 |
| miR-296 | 1.62 | 2.81E-02 |
| miR-199b | 3.47 | 2.82E-02 |
| miR-518c | 1.97 | 2.84E-02 |
| miR-216 | 1.46 | 3.49E-02 |
| miR-649 | 1.82 | 3.90E-02 |
| miR-621 | 6.64 | 4.11E-02 |
| miR-452 | 1.60 | 4.27E-02 |
| miR-550 | 1.95 | 4.63E-02 |
| miR-200b | 1.78 | 4.96E-02 |
| miR-509 | -1.53 | 6.31E-03 |
| miR-518d-5p | -1.65 | 1.11E-02 |
| miR-556 | -1.60 | 3.26E-02 |
| miR-21 | -4.53 | 3.59E-02 |
| miR-422a | -1.57 | 3.99E-02 |
| miR-557 | -3.02 | 4.70E-02 |
| miR-520h | -6.51 | 4.90E-02 |

**Table S5.** MicroRNAs differentially expressed (*P* < 0.05) between IgA (n = 8) and IgG (n = 13) isotype MM cases. Positive values up-regulated in IgG-isotype.

| **microRNA** | **Fold change** | ***P*-value** |
| --- | --- | --- |
| miR-521 | -2.14 | 6.79E-04 |
| miR-486-3p | -2.07 | 3.00E-02 |
| miR-553 | -2.02 | 1.78E-02 |
| miR-452* | -2.01 | 1.02E-02 |
| miR-628-3p | -1.96 | 2.62E-02 |
| miR-620 | -1.90 | 4.62E-02 |
| miR-566 | -1.86 | 2.32E-02 |
| miR-892a | -1.81 | 1.15E-02 |
| miR-339-5p | -1.81 | 2.98E-02 |
| miR-628 | -1.78 | 2.14E-02 |
| miR-421 | -1.77 | 3.26E-02 |
| miR-520d-5p | -1.76 | 3.09E-02 |
| miR-616 | -1.73 | 4.44E-02 |
| miR-297 | 1.58 | 3.42E-02 |
| miR-213 | 1.69 | 3.07E-02 |
| miR-519e* | 1.97 | 9.59E-03 |
| miR-422a | 2.09 | 1.78E-02 |
| miR-183 | 2.16 | 9.88E-03 |
| miR-198 | 2.27 | 2.21E-03 |
| miR-122a | 2.38 | 4.97E-02 |
| miR-1236 | 2.46 | 1.28E-02 |

**Table S6.** MicroRNAs differentially expressed (*P* < 0.05) between LC-only myeloma (n = 8) and non-LC-only myeloma MM cases (n = 21). Positive values are up-regulated in LC-only cases.

| **microRNA** | **Fold change** | ***P*-value** |
| --- | --- | --- |
| miR-548c-5p | -2.41 | 2.04E-03 |
| miR-191* | -2.23 | 1.83E-02 |
| miR-583 | -2.20 | 3.65E-02 |
| miR-188-5p | -2.05 | 6.35E-03 |
| miR-376c | -2.02 | 2.06E-02 |
| miR-532-3p | -1.90 | 3.29E-02 |
| miR-34c-3p | -1.82 | 4.27E-02 |
| miR-122a | -1.82 | 2.32E-02 |
| miR-453 | -1.68 | 1.43E-02 |
| miR-509 | -1.53 | 3.48E-02 |
| miR-124a | 1.55 | 1.24E-02 |
| miR-516-3p | 1.56 | 3.19E-02 |
| miR-505 | 1.60 | 1.38E-02 |
| miR-18a | 1.69 | 2.28E-02 |
| miR-208 | 1.69 | 2.21E-02 |
| miR-659 | 1.71 | 4.59E-03 |
| miR-146b | 1.88 | 3.22E-02 |
| miR-518c | 1.88 | 7.44E-03 |
| miR-665 | 1.90 | 1.13E-03 |
| miR-485-5p | 2.08 | 3.57E-02 |
| miR-146a | 2.53 | 2.01E-02 |
| miR-128a | 2.59 | 3.62E-02 |
| miR-378 | 2.70 | 1.17E-02 |
| miR-191 | 2.83 | 3.93E-02 |
| miR-324-5p | 3.04 | 2.83E-02 |
| miR-371-3p | 3.67 | 8.80E-03 |
| miR-152 | 3.94 | 1.96E-02 |

**Table S7.** MicroRNAs associated with event-free survival (EFS) in MM cases. ANOVA analysis (*P* < 0.05) was carried out between cases that were event-free (n = 7) and those with events (relapse or death) (n = 21). MicroRNAs that were significantly (*P*<0.05) associated with EFS by univariate (logrank) analysis using median expression levels as a cut-off are highlighted in bold type. Positive fold change is associated with event and negative fold change event-free status.

| **microRNA** | **Fold change** | ***P*-value** | ***P*-value (univariate)** |
| --- | --- | --- | --- |
| hsa-miR-507 | 2.48 | 6.93E-03 | 0.09 |
| hsa-miR-302b* | 2.18 | 1.27E-02 | 0.10 |
| **hsa-miR-373** | 2.18 | 2.79E-03 | **0.01** |
| hsa-miR-569 | 2.12 | 6.45E-03 | 0.07 |
| hsa-miR-491-5p | 2.11 | 3.43E-02 | 0.32 |
| **hsa-miR-548d** | 1.96 | 1.14E-02 | **0.04** |
| **hsa-miR-888** | 1.96 | 3.79E-02 | **0.02** |
| **hsa-miR-153** | 1.91 | 2.56E-02 | **0.004** |
| **hsa-miR-554** | 1.85 | 1.69E-02 | **0.01** |
| hsa-miR-596 | 1.84 | 2.66E-02 | 0.18 |
| hsa-miR-520c | 1.83 | 6.69E-03 | 0.17 |
| hsa-miR-519b-3p | 1.81 | 1.47E-02 | 0.09 |
| hsa-miR-410 | 1.80 | 3.28E-02 | 0.05 |
| hsa-miR-371-5p | 1.77 | 4.55E-02 | 0.39 |
| hsa-miR-564 | 1.76 | 1.56E-02 | 0.45 |
| hsa-miR-384 | 1.75 | 4.34E-02 | 0.21 |
| hsa-miR-302a* | 1.66 | 4.57E-02 | 0.11 |
| hsa-miR-617 | 1.65 | 3.48E-02 | 0.23 |
| hsa-miR-492 | 1.58 | 4.92E-02 | 0.13 |
| hsa-miR-193a-3p | 1.52 | 2.78E-02 | 0.09 |
| hsa-miR-302a | 1.40 | 4.49E-02 | 0.13 |
| **hsa-miR-642** | -1.63 | 3.63E-02 | **0.02** |
| hsa-miR-575 | -1.79 | 4.23E-02 | 0.08 |
| hsa-miR-194 | -1.82 | 3.39E-02 | 0.06 |
| **hsa-miR-490** | -1.96 | 4.53E-04 | **0.008** |
| **hsa-miR-296** | -1.97 | 3.60E-02 | **0.04** |
| hsa-miR-532-3p | -1.97 | 1.10E-02 | 0.06 |
| **hsa-miR-455-3p** | -2.00 | 2.37E-02 | **0.01** |
| hsa-miR-323 | -2.27 | 4.70E-02 | 0.08 |
| hsa-miR-454 | -2.30 | 2.32E-02 | 0.34 |
| **hsa-miR-500** | -2.32 | 4.76E-02 | **0.01** |
| hsa-miR-515-3p | -3.27 | 3.25E-02 | 0.12 |


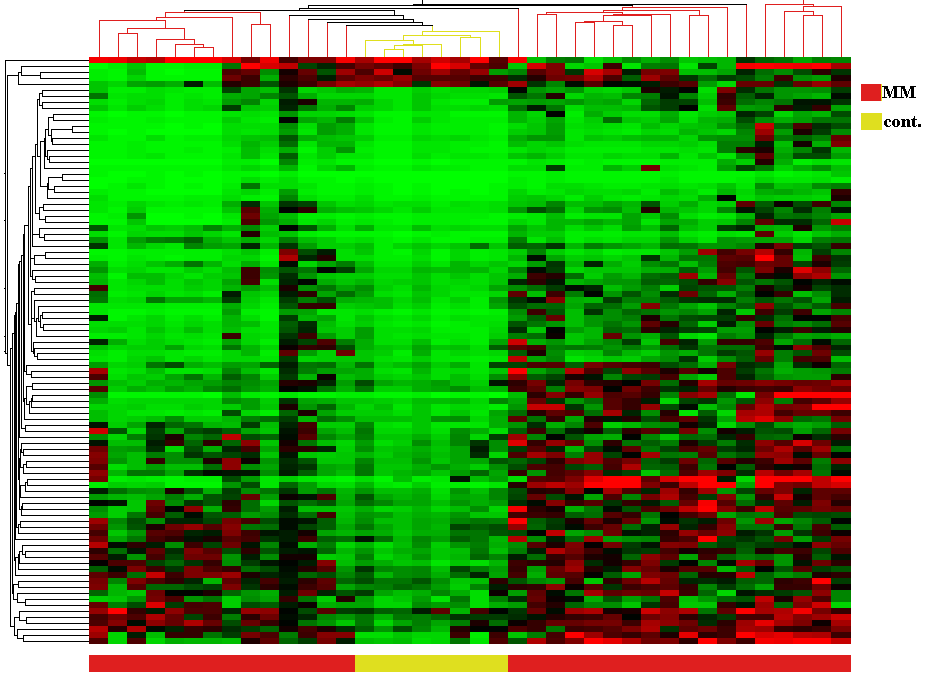


**Figure S1**. Heat map depicting cluster analysis of MM and control samples on the basis of expression values of 129 MM-associated microRNAs (Table 1).


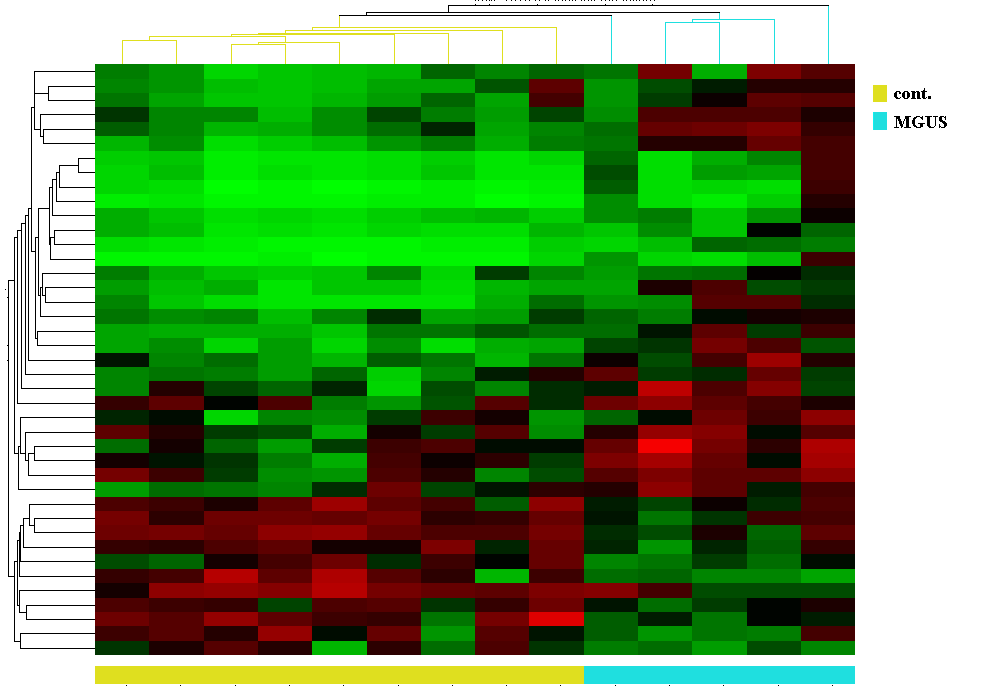


**Figure S2**. Heat map depicting cluster analysis of MGUS and control samples on the basis of expression values of 39 MGUS-associated microRNAs (Table S3).


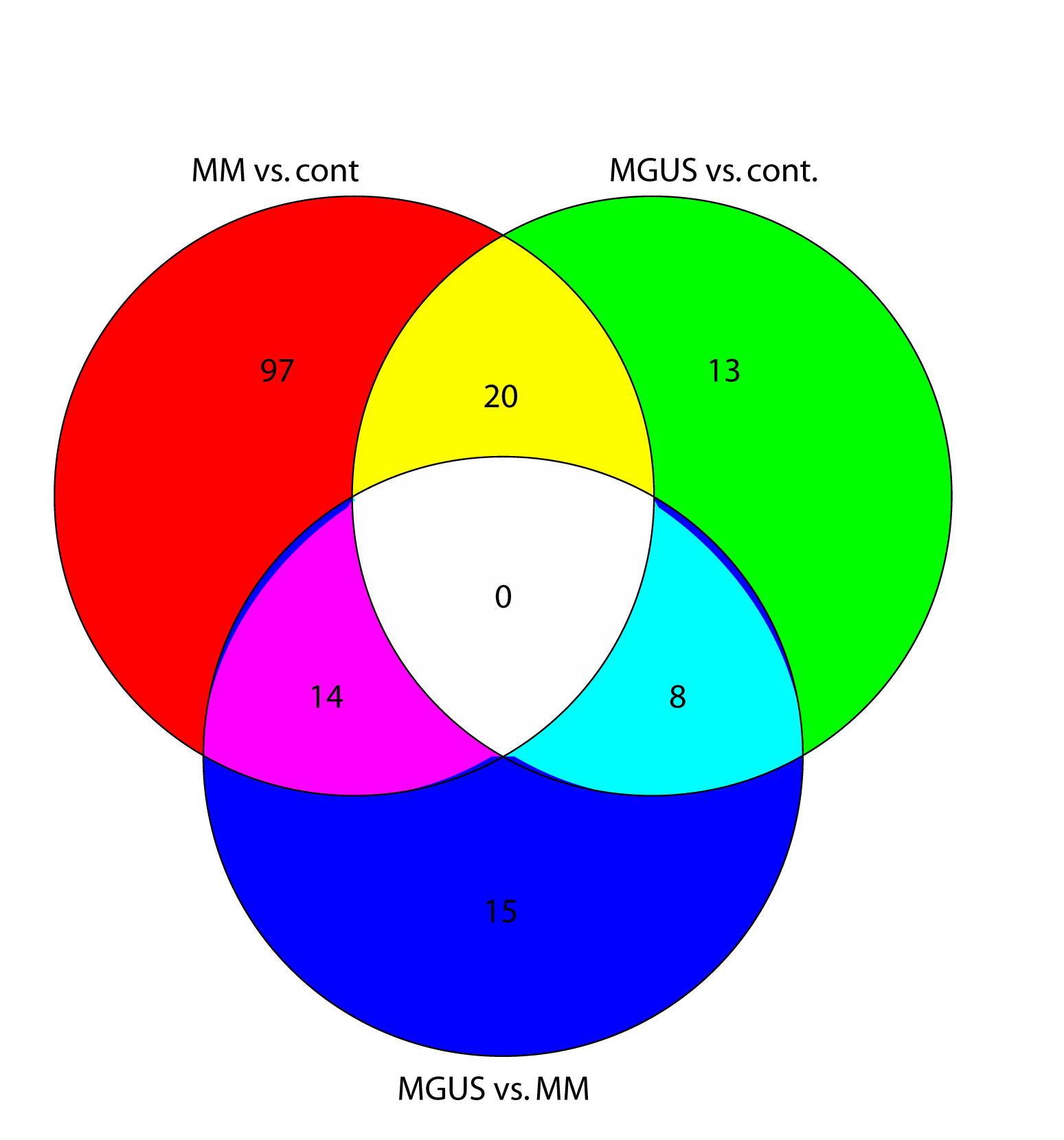


**Figure S3.** Venn-diagram depicting relationship between microRNAs differentially expressed in MGUS vs. controls (Table S3), MGUS vs. MM (Table S4, and MM vs. controls (Table 1).


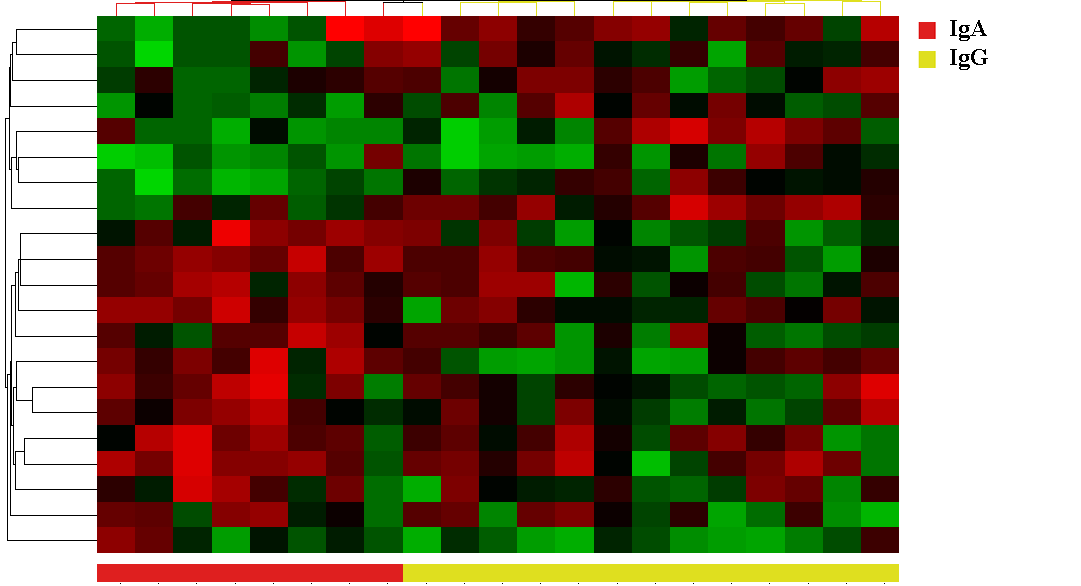


**Figure S4.** Heat map depicting cluster analysis of IgG and IgA isotype MM cases on the basis of expression values of 21 isotype-associated microRNAs (Table S5).
